# *Trypanosoma brucei* histones are heavily modified with combinatorial post-translational modifications and mark Pol II transcription start regions with hyperacetylated H2A

**DOI:** 10.1093/nar/gkac759

**Published:** 2022-09-12

**Authors:** Johannes P Maree, Andrey Tvardovskiy, Tina Ravnsborg, Ole N Jensen, Gloria Rudenko, Hugh-G Patterton

**Affiliations:** Department of Biochemistry, Stellenbosch University, Stellenbosch 7600, South Africa; Department of Biochemistry and Molecular Biology, VILLUM Center for Bioanalytical Sciences, and Center for Epigenetics, University of Southern Denmark, Odense M DK-5230, Denmark; Department of Biochemistry and Molecular Biology, VILLUM Center for Bioanalytical Sciences, and Center for Epigenetics, University of Southern Denmark, Odense M DK-5230, Denmark; Department of Biochemistry and Molecular Biology, VILLUM Center for Bioanalytical Sciences, and Center for Epigenetics, University of Southern Denmark, Odense M DK-5230, Denmark; Department of Life Sciences, Imperial College London, London SW7 2AZ, UK; Center for Bioinformatics and Computational Biology, Stellenbosch University, Stellenbosch 7600, South Africa

## Abstract

*Trypanosomes* diverged from the main eukaryotic lineage about 600 million years ago, and display some unusual genomic and epigenetic properties that provide valuable insight into the early processes employed by eukaryotic ancestors to regulate chromatin-mediated functions. We analysed *Trypanosoma brucei* core histones by high mass accuracy middle-down mass spectrometry to map core histone post-translational modifications (PTMs) and elucidate *cis*-histone combinatorial PTMs (cPTMs). *T. brucei* histones are heavily modified and display intricate cPTMs patterns, with numerous hypermodified cPTMs that could contribute to the formation of non-repressive euchromatic states. The *Trypanosoma brucei* H2A C-terminal tail is hyperacetylated, containing up to five acetylated lysine residues. MNase-ChIP-seq revealed a striking enrichment of hyperacetylated H2A at Pol II transcription start regions, and showed that H2A histones that are hyperacetylated in different combinations localised to different genomic regions, suggesting distinct epigenetic functions. Our genomics and proteomics data provide insight into the complex epigenetic mechanisms used by this parasite to regulate a genome that lacks the transcriptional control mechanisms found in later-branched eukaryotes. The findings further demonstrate the complexity of epigenetic mechanisms that were probably shared with the last eukaryotic common ancestor.

## INTRODUCTION

*Trypanosoma brucei* is a flagellated parasitic protozoan that causes human African trypanosomiasis and Nagana in livestock, and is transmitted to the mammalian host during a blood meal by a tsetse fly of the *Glossina* genus (1). As the parasite cycles between the mammalian host and the insect vector, it differentiates into different life cycle stages including the mammalian infective bloodstream form (BF) and the procyclic form (PF) in the midgut of the tsetse fly (2). These life-cycle transitions are accompanied by major changes in metabolic pathways, mitochondrial activity, and gene expression programs. Although the number of yearly reported infections has steadily declined over the past decade, it is estimated that there are ∼60 million people at risk of contracting this disease in sub-Saharan Africa (3,4). This number may increase drastically by 2040 due to a change in the vector distribution footprint brought on by global warming (3,5).

It is generally accepted that the last eukaryotic common ancestor (LECA) arose some 1.8 billion years ago (6). The subsequent expansion in cellular complexity was typically associated with an increase in genetic information encoded in DNA, which, in turn, required more DNA to fit into the relatively small nucleus (7). This extreme degree of DNA compaction is accomplished by spooling ∼146 bp of DNA in two negative supercoils onto a histone octamer to condense the polyanionic DNA and form a nucleosome, where arrays of nucleosomes are packaged into higher-order levels of folding. The nucleosome is the basic structural unit of chromatin, and an ancient evolutionary adaptation shared between Archaea and Eukarya (8,9).

The *Excavata* superclade, which contains the order *Trypanosomatida*, separated from the main eukaryotic lineage some 600 million years ago (10,11). It is conceivable that because of this early divergence, trypanosomes lack many of the conserved transcription factors identified in later-branched eukaryotes (12). In addition, they exhibit some unusual genomic characteristics such as the relative lack of clearly identifiable RNA polymerase II (Pol II) promoter and other regulatory DNA sequences, and there is constitutive genome-wide polycistronic Pol II-mediated gene transcription. There is also highly unusual transcription of protein-coding genes by Pol I (13–15).

The Pol II transcribed region of the *T. brucei* genome is arranged as long, non-overlapping, polycistronic transcription units (PTUs) separated by strand switch regions (SSRs) (16). During mRNA maturation, a 39 nt spliced leader RNA (SL RNA) sequence is trans-spliced to the 5′ end of each protein-coding transcript. The SL RNA arrays, located on chromosome 9, are transcribed at high rates by Pol II, and these high levels of SL RNA expression ensure that it is not a rate-limiting factor in mRNA maturation (17–19). Transcription of trypanosomal PTUs can initiate either at divergent SSRs (dSSRs) between divergently transcribed gene clusters or, alternatively, at internal start sites (ISSs) where tandem gene clusters are arranged head-to-tail (20,21).

*T. brucei* relies to a great extent on its epigenome to carry out chromatin-mediated regulation achieved through deposition of histone variants and histone post-translational modifications (PTMs), which can confer structural changes in chromatin to delineate and control genomic function (19,22,23). Transcription initiation regions at the dSSRs are marked by nucleosomal incorporation and acetylation of H2A.Z and H2B.V histone variants, as well as deposition of H3K4me3 and H4K10ac, among other epigenetic marks (21,24,25). A recent systematic analysis of *T. brucei* chromatin factors found that 15 chromatin reader proteins, including six bromodomain proteins (BDFs) and three histone acetyl transferases (HATs), were enriched at putative Pol II transcription start regions (26). Pol II transcription terminates at convergent SSRs (cSSRs), marked by H3.V and H4.V histone variants, and at tandem head-to-tail PTUs where Pol II transcription needs to terminate and then reinitiate (21). Transcriptional termination is also influenced by the presence of an rRNA or tRNA gene that presents a steric hindrance to a transcribing Pol II complex (21).

Histones are among the most conserved proteins in eukaryotes. However, *T. brucei* histones diverge sharply from those of other eukaryotes, especially in the N- and C-terminal tail regions (27). This divergence makes trypanosomal epigenetic processes an attractive target for the development of epigenetic therapies to treat sleeping sickness. Indeed, it was demonstrated that epigenetic therapeutics targeting histone and non-histone epigenetic modifiers in *Plasmodium falciparum* showed transmission-blocking potential (28).

Although trypanosomes diverged very early from the main eukaryotic lineage, they do possess the fundamental chromatin-modifying machinery. Previous studies revealed the presence of 173 PTMs on canonical and variant histone isoforms in *T. brucei* (25,29–34). A recent study that employed a novel approach to identify Pol II transcription start site (TSS) and non-TSS specific core histone PTMs found that nucleosomes at TSS are highly acetylated (25). TSS-associated nucleosomes were found to contain hyperacetylated H2A.Z and H2B.V, and N-terminally modified H3 and H4 histones which were methylated or acetylated to varying degrees. Almost no acetylation was detected on histone H3.V and H4.V variants (25).

Histone PTMs do not necessarily act in a singular fashion to produce a functional readout but can form intricate combinatorial PTM (cPTM) patterns. These can act in cooperation by modulating the functions of one another and ‘fine-tune’ chromatin-mediated regulation by recruitment of different chromatin remodelers, or through the disruption of existing intra- and inter-molecular interactions (22,23). Therefore, to understand the regulatory roles of histone PTMs, it is critically important to elucidate their combinatorial complexity. The existence of elaborate histone cPTMs has been demonstrated in a range of eukaryotes, where they influence gene expression, biological functions, ageing and disease states (35–38). A study by Mandava and colleagues indicated the presence of cPTMs on short tryptic histone peptides in *T. brucei*, where multiple acetylation marks were observed on C-termini of H2A peptides (31). These data point toward the existence of cPTM patterns on *T. brucei* histones where neither the combinatorial histone modification repertoire nor the functional importance is known.

To investigate the epigenetic histone repertoire of *T. brucei*, we undertook a middle-down tandem mass spectrometric (MS) analysis of acid extracted core histone isoforms. This technique relies on the LC-MS/MS analysis of long peptides that enables unambiguous mapping of multiple co-existing PTMs present on the same histone molecule (37). Our analyses revealed 203 individual PTMs, of which 94 are novel, and identified 110 distinct combinatorial PTM patterns. Analysis of the genomic distribution of hyperacetylated H2A cPTMs revealed a striking enrichment at Pol II TSSs, suggesting a link between cPTM deposition and transcription. This is, to the best of our knowledge, the first time cPTMs have been studied in *T. brucei*, and suggests that the utilization of histone cPTMs pre-dates the split between *Excavata* and *Unikonts*.

## MATERIALS AND METHODS

### Trypanosome strains and culture

BF *T. brucei brucei* 427 *SmoxB4* was cultured *in vivo* in rats (39). For core histone extraction from BF trypanosomes, Swiss Albino rats were inoculated intra-peritoneally with 1 × 10^4^ trypanosomes, and monitored for parasitemia post-infection using the Herbert and Lumsden matching method (40). At the parasitemia peak (10^8^ cells/ml) animals were exsanguinated, and blood collected by cardiac puncture into heparinised vacutainers, yielding 8–10 ml blood/animal. Trypanosomes were purified using DEAE anion exchange chromatography yielding ∼9 × 10^10^ parasites per sample (41). PF *T. brucei* 427 cells were cultured at 27°C in SDM-79 medium (SDM-79, 2 g/l; NaHCO_3_, pH 7.3), supplemented with 10% v/v fetal calf serum (FCS) and 5 μg/ml hemin (42).

For MNase-ChIP-seq, BF *T. brucei* (S16 221 Puro) was cultured in HMI-9 medium supplemented with 15% v/v FCS, 0.1 mM β-mercaptoethanol, and 0.2 μg/ml puromycin at 37°C with 5% CO_2_ as previously described (43). PF trypanosomes were cultured as described above.

### Core histone isolation

Core histones were extracted using an acid extraction protocol adapted for *T. brucei* (44). In brief, 1 × 10^10^ and 1 × 10^9^ cells/sample were harvested for BF (*n* = 11) and PF (*n* = 9) trypanosomes, respectively. Cells were resuspended in 1 ml Buffer A (0.25 M sucrose; 1 mM EDTA; 3 mM CaCl_2_; 10 mM Tris–HCl pH 7.4; 0.5% v/v saponin), and centrifuged for 10 min at 4000 RCF, 4°C. The pellet was resuspended in 1 ml Buffer B (0.25 M sucrose; 1 mM EDTA; 3 mM CaCl_2_; 10 mM Tris–HCl pH 7.4), and centrifuged for 10 min at 4000 RCF, 4°C. The resulting pellet was resuspended in 1 ml Buffer C (1% v/v Triton X-100; 0.15 M NaCl; 25 mM EDTA; 10 mM Tris–HCl pH 8.0), and centrifuged for 20 min at 12 000 RCF, 4°C. The pellet was washed three times in 1 ml 100 mM Tris–HCl (pH 8.0) and pelleted at 12 000 RCF, 4°C for 5 min. The chromatin pellet was resuspended in 600 μl of 0.4 N H_2_SO_4_. An Eppendorf Dounce homogenizer was used to solubilize histones from chromatin, and samples incubated at 4°C for 6 h under rotation. Acid-soluble proteins were recovered by centrifugation at 10 000 RCF for 15 min, 4°C. The supernatant volume was determined (∼500 μl per sample), transferred to a fresh tube, and trichloroacetic acid (TCA) added to a final concentration of 33% (w/v). Samples were incubated overnight at 4°C under gentle agitation and pelleted at 16 000 RCF, 10 min, 4°C. The pellet was washed three times with ice-cold (−20°C) acetone and centrifuged at 16 000 RCF, 5 min, 4°C. Pellets were air dried for 10 min, and stored at −80°C. Protease inhibitors were added to all buffers just before use and included 0.1 mM PMSF, 10 mM sodium butyrate, protease inhibitor cocktail (cOmplete mini, EDTA-free, Roche), and phosphatase inhibitors (Halt Protease and Phosphatase Inhibitor Cocktail, Thermo Scientific) per the manufacturer's instructions.

### Proteolytic digestion

Histone pellets were resuspended in 50 μl ddH_2_O by sonication (5 min) followed by agitation under rotation for 1 h at 4°C. This was done four times for a total of four hours, and insoluble debris pelleted at 16 000 RCF for 10 min, 4°C. Protein concentrations were determined by the BCA method with a BCA Protein Assay Kit (Thermo Scientific). For protein digestion and middle-down MS analysis, a total of 30 μg protein/sample was diluted with 75 mM ammonium acetate (pH 4.0) to a final concentration of 1 μg/μl. Proteolytic digestion was performed using Glu-C or Asp-N endoproteinases (Calbiochem) with an enzyme:sample ratio of 1:20 and 1:40, at room temperature and 37°C, for 6 h and overnight, respectively.

### Nano-LC MS/MS

Peptides were separated using a nanoliter-flow Ultimate 3000 HPLC system (Thermo Scientific) as previously described (37). The nano-LC was equipped with a two-column set-up comprised of a 5 cm pre-column (100 μm internal diameter) packed with C_18_ bulk material (ReproSil, Pur C18AQ 5 μm), and an 18 cm analytical column (75 μm internal diameter) packed with PolycatA resin (3 μm particles, 1500 Å; PolyLC, Columbia, MD, USA). Sample loading buffer contained 0.1% (v/v) formic acid in ddH_2_O. Buffers A and B were prepared as previously described (36). Peptides were separated using a gradient of 100% Buffer A (75% v/v acetonitrile (ACN); 20 mM propionic acid; pH 6.0) for 10 min, followed by 30–95% Buffer B (25% v/v ACN; pH 2.5) for 105 min, and 95–100% Buffer B (10 min) for column washing. The flow rate for analysis was set to 230 nl/min. The nano-LC was coupled in line with an Orbitrap Fusion ETD MS controlled by Xcalibur software (Thermo Scientific). A nano-electrospray ion source was used with spray voltage set at 2.4 kV and capillary temperature set at 275°C. Data acquisition was performed in the Orbitrap instrument for precursor and product ions, with a mass resolution of 60 000 for MS and 30 000 for MS/MS. The MS acquisition window was set at *m/z* 400–750 with dynamic exclusion disabled. Precursor ion charges for MS/MS fragmentation was set to 5–12, and isolation width was set at 2 *m/z* units. The six most intense precursor ions (MS signal > 5000 counts) were isolated for ETD MS/MS fragmentation, with an activation time of 20 ms. Three microscans were used for each MS/MS spectrum, with automatic gain control target set to 2 × 10^5^.

### Middle-down MS data processing and analysis

MS data files were processed with Proteome Discoverer (v2.4, Thermo Scientific), spectra deconvoluted with the Xtract tool (Thermo Scientific) and searched using Mascot (v2.7.0.1, Matrix Science, London, UK). Mascot searches were carried out with the following parameters: MS peptide mass tolerance: 1.05 Da; MS/MS fragment mass tolerance: ±0.015 Da; enzyme: Glu-C or Asp-N with no missed cleavages; Mass values: monoisotopic; Dynamic modifications: N-terminal acetylation, mono- and di-methylation (K, R), tri-methylation (K), acetylation (K), oxidation (P,M,W,Y), and phosphorylation (T,S). To search the MS/MS spectra, canonical and variant histone protein sequences encoded in the *T. brucei* 427 genome were retrieved from TriTryDB (v25, http://tritrypdb.org/tritrypdb/) and used to construct a custom protein database that included (protein: GeneID): H2A: Tb427.07.2830; H2A.Z: Tb427.07.6360; H2B: Tb427.10.10460; H2B.V: Tb427tmp.02.5250; H3: Tb427.01.2430; H3.V: Tb427.10.15350; H4: Tb427.05.4170; and H4.V: Tb427.02.2670.

### PTM site validation, quantitation, spectral visualization and alignments

Mascot search results were exported to an XML format file and processed with Histone Coder using a tolerance of 30 ppm. Only PTMs with at least one bordering site determining ion were accepted for further analysis (37). Mascot search results were exported to a DAT format file. Peptide spectrum matches verified by Histone Coder were generated and visualized using the Mascot Spectrum viewer. Mass spectra of newly identified PTMs are shown in Supplementary Tables S1–S8. For the canonical histones identified, the percentage of modified peptides as compared to unmodified peptides was estimated for selected representative samples. For each sample an average MS1 spectrum was created across the retention time 30–160 min (Thermo Scientific FreeStyle), and the relevant MS1 peaks were then identified for the major assigned proteoforms, based on the *m*/*z* and the characteristic pattern of 14.02 Da/*z* increments. The percentage of each major proteoform relative to the total amount of major proteoforms for each peptide was calculated by using the signal to noise (S/N) value for each peak (Supplementary Tables S1, S3, S5, S7). Applying the same type of calculations for the sum intensity of the deconvoluted average MS1 spectrum (Xtract) gave similar results. Sequence alignments for core histones from *Trypanosoma brucei, Trypanosoma cruzi, Homo sapiens, Saccharomyces cerevisiae, Schizosaccharomyces pombe, Drosophila melanogaster* and *Mus musculus* are provided on sheet 1 of Supplementary Tables S1, S3, S5 and S7. Sequence alignments were performed using Clustal Omega (45).

### Antibody generation

Polyclonal antibodies were raised in New Zealand white rabbits against histone H3 and hyperacetylated H2AK115ac and H2AK125ac using K_16_KSKKASKGSDAAS_29_C, L_113_NK_115ac_ALAKK_120ac_QK_122ac_SGKHAC and L_113_NKALAK*K_120ac_*Q*K_122ac_*SG*K_125ac_*HAC synthetic peptides, respectively (GenScript Incorporated, Piscataway, USA). Antibodies were affinity purified and the titer, specificity, and cross-reactivity determined via indirect ELISA (Supplementary Table S9, sheet1) (GenScript Incorporated, Piscataway, USA). Antibody specificity was verified against an unmodified LNK_115_ALAKK_120_QK_122_SGK_125_HAC peptide, as well as hyperacetylated peptides. Further information on hyperacetylated H2A antibody generation, synthetic peptide sequences used to raise antibodies, indirect ELISAs performed, as well as antigenic plots are shown in Supplementary Table S9, sheet 1.

### Peptide-based competition assay and dot blots

Histones were acid extracted as described above, 5 μg total protein run on a 10% SDS-PAGE and transferred to PVDF membrane. Antibodies were incubated with unmodified or differentially modified peptides for 1 h in 1% blocking buffer at room temperature with gentle agitation. Western blotting was carried out according to standard protocols. The following primary antibodies were used: α-H2AK125ac (1:1000), α-H2AK115ac (1:1000) and α-H3 (1:5000). Goat anti-rabbit horseradish peroxidase coupled secondary antibodies were used, developed using Clarity Western ECL substrate (Bio-Rad). For dot-blot analysis, 5 μg unmodified or differentially modified peptides were spotted onto a PVDF membrane and probed with either α-H2AK125ac or α-H2AK115ac antibodies and developed using a Pierce Fast Western Blot kit, according to manufacturer's instructions. Anti-histone H3 was used as loading control (Abcam ab1791).

### MNase-ChIP-seq

A native ChIP (N-ChIP) approach was applied as previously described (44). In brief, 2.5 × 10^8^ cells per sample were harvested and chromatin digested with 4 U MNase (Worthington Biochemicals) at 37°C for 5 min. Digested chromatin was diluted 10 times using ChIP dilution buffer (0.01% SDS; 1.1% v/v Triton X-100; 1.2 mM EDTA; 16.7 mM Tris–HCl, pH 8.1), and 200 μl retrieved from each sample as input DNA. The solution was precleared with 60 μl protein A coupled magnetic beads (Dynabeads, Invitrogen) whereafter 10 μg antibody (α-H3: *n* = 1 BF and PF; α-H2AK115ac: *n* = 1 BF and PF; α-H2AK125ac: *n* = 2 BF and PF) was added to the supernatant and incubated overnight. Following immune-coupling, 80 μl Dynabeads was added and incubated for 2 h at 4°C to collect the antibody–protein–DNA complex. Dynabeads were collected using a magnetic rack, the supernatant aspirated, and washed three times each with 1 ml low-salt buffer (0.1% SDS; 1% v/v Triton X-100; 2 mM EDTA; 20 mM Tris–HCl pH 8.1; 150 mM NaCl), followed by high-salt buffer (0.1% SDS; 1% v/v Triton X-100; 2 mM EDTA; 20 mM Tris–HCl pH 8.1; 500 mM NaCl), LiCl wash buffer (0.25 M LiCl; 1% v/v NP-40; 1% deoxycholate; 1 mM EDTA; 500 mM NaCl; 10 mM Tris–HCl pH 8.1) and finally six times with TE buffer (1 mM EDTA; 10 mM Tris–HCl pH 8). The DNA–protein complex was eluted with 400 μl elution buffer (1% SDS; 0.1 M NaHCO_3_). Input DNA samples were diluted with 10 mM Tris–HCl (pH 8.0) to a final volume of 400 μl. DNA was released from the histone octamer by proteinase K digestion and phenol:chloroform extraction, followed by ethanol precipitation. DNA fragments were sequenced using 125 nt paired-end methodology (Illumina) as previously described (46).

### Bioinformatics data analysis

*Trim_Galore!* was used for quality trimming, adapter clipping, and quality control with minimum and maximum read lengths set to 115 and 125 bp, respectively (47). *FastQC* was used to assess sequence quality after trimming, and trimmed reads re-aligned to the *T. brucei* 427 genome (v37, http://tritrypdb.org/tritrypdb/), with Bowtie 2 using default settings (48,49). The Bowtie 2 alignment files were analyzed for read enrichment using MACS2 with a user-defined genome size (26.75 Mb); 200 nt fragment size for shifting model; minimum false discovery rate: ≥0.01; composite broad regions: true (50). Bowtie 2 as well as MACS2 output files were visualized with Integrated Genome Viewer (IGV) (51,52). A graphical representation of the workflow is given in Supplementary Figure S1. Bioinformatics analyses was done on the Galaxy platform (53). The workflow is publicly listed and searchable in Galaxy's published workflows section (‘reads to peaks’), and can be accessed at https://usegalaxy.eu/u/jpm/w/reads-to-peaks. Data on NGS sequence reads and realignment are shown in Supplementary Table S9, sheet 2. Heatmaps were generated using deeptools (54). For SSR heatmaps, reads were aligned in 10 kb regions up- and downstream relative to the center of the SSRs. For ISS heatmaps, regions were grouped based on strandedness and aligned in 10 kb regions up- and downstream relative to the ISS start.

## RESULTS

### Identification of core histone post-translational modifications

**Figure 1.**
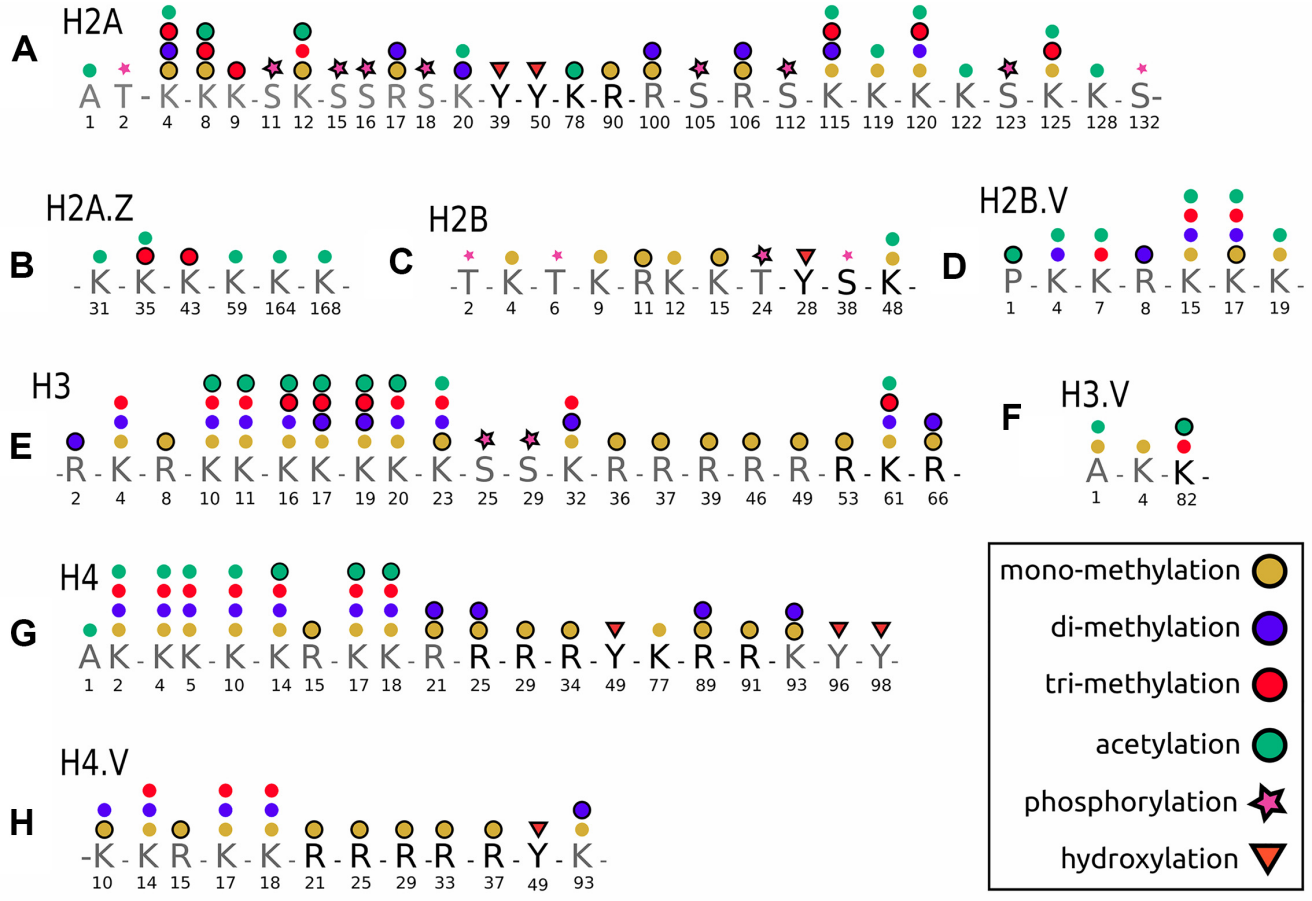
Schematic overview of post-translational modifications identified on canonical and variant core histones in *T. brucei*. (**A**) H2A, (**B**) H2A.Z, (**C**) H2B, (**D**) H2B.V, (**E**) H3, (**F**) H3.V, (**G**) H4, (**H**) H4.V. All histone PTMs were identified using middle-down mass spectrometric analysis, and validated using Histone Coder. Lettering in light grey indicates histone tails while letters in black indicate histone fold regions. Different symbols indicate the histone modifications, with those marked with black outlines indicating novel PTMs identified in this study. Supplementary Tables S1–S8 provide mass spectra of all PTMs identified in this study, while Supplementary Table S9 sheet 3 shows PTMs identified in the present and past studies.

Acid extraction of nuclear proteins allows the enrichment of core histones with minimal contamination from other major nuclear molecules (55). This facilitates extraction of all core histone isoforms regardless of their genomic distribution. Aiming at a comprehensive characterization of the *T. brucei* histone modification landscape, including combinatorial PTMs, we first optimized a histone protein digestion strategy that would allow for the generation of long histone peptides. To this end, we took advantage of proteolytic digestion of histones with endoproteinase Glu-C or Asp-N which produces large peptides of 21–69 amino acids in length (Supplementary Tables S1–S8, sheet 1). These long peptides are amenable to middle-down mass spectrometric sequencing and mapping of single and combinatorial PTMs (37). The differentially modified histone peptide proteoforms were then separated using weak cation exchange-hydrophilic interaction chromatography (WXC-HILIC) and fragmented using electron-transfer dissociation (ETD). Application of this approach on BF and PF *T. brucei* acid extracted histones identified 203 PTMs, of which 109 were previously described and 94 novel modifications were identified. These included 66 mono-, 41 di-, 34 tri-methylation, 39 acetylation, 16 phosphorylation and 7 hydroxylation marks (Figure 1). Previously identified PTMs not detected by our analysis are indicated in Supplementary Table S9, sheet 3.

### Modifications of *T. brucei* histone H2A isoforms

Histone H2A was the most densely modified of the eight core histone isotypes. We mapped a total of 48 PTMs to H2A, of which 31 are newly identified (Figure 1A). The majority of H2A modifications were observed on the N- and C-terminal tails, although four were mapped to residues in the globular domain. H2A N-terminal lysines K4, K8, K9, K12 and K20 were modified to various degrees with acetylation, mono-, di- or tri-methylation marks. The majority of the N-terminal H2A modifications have not been previously documented for these H2A lysine residues in *T. brucei*, with the exception of K4ac, K12me3 and K20ac (25,31,34). Three of these N-terminal H2A lysines align with known sites of H2A modification in other eukaryotes. *T. brucei* H2A K4 aligns with *H. sapiens* H2A K5 and *S. cerevisiae* H2A K4. These lysine residues are associated with transcriptional activation in their acetylated states, which is mediated by the HAT1 acetyltransferase (56,57). *T. brucei* H2A K12 aligns with *S. cerevisiae* and *H. sapiens* H2A K13 where it has been observed to be either mono-methylated or acetylated (56). Arginine 17 displayed mono- and di-methylation modifications which have not previously been observed at this residue in trypanosomes. We observed multiple phosphorylation marks on the N-terminus of H2A at positions T2, S11, S15, S16 and S18. These polar amino acids are located on the side of the nucleosomal disk and are involved in protein folding (8). Phosphorylation of these residues may alter chromatin structure by interfering with tertiary structure formation, similar to those observed at the H2A C-terminal serine and threonine residues (58).

Hydroxylation of tyrosine residues in histones has been observed in a range of eukaryotes, from humans to the related *T. cruzi*. In *T. brucei*, we detected tyrosine hydroxylation at H2A Y39 and Y50 globular domain residues, one of which (Y50) forms part of a hydrophobic pocket associated with the acidic patch (59). The acidic patch is an electronegative cleft formed by seven glutamic acid and one aspartic acid residue on the histone H2A–H2B dimer and creates a binding interface on the nucleosomal surface (8). Three additional H2A residues (Y50, V54 and Y57) form a hydrophobic pocket that contributes to inter-nucleosomal interactions to form higher-order chromatin structures (59). The overall hydrophobicity of tyrosine residues is somewhat tempered by the polar hydroxy group on carbon 4 (60). Additional hydroxylation, as observed on *T. brucei* H2A Y50, could further mitigate the relative hydrophobicity of the hydrophobic pocket and may affect inter-nucleosomal interaction facilitated by the acidic patch.

We also observed K78 and R90 in acetylated and mono-methylated forms in the globular domain of H2A, respectively. TbR90 aligns with human R88, which was found to be mono-methylated. Methylation of this arginine residue will perturb the p*K*a of the side chain and influence the histone:DNA interactions (61,62). We detected mono- and di-methylation of R100 and R106 on *T. brucei* H2A, but these residues do not appear to have orthologous residues in other eukaryotes.

The H2A C-terminal tail contains multiple lysine residues that can interact with DNA near the nucleosome termini (8,63). We found that H2A C-terminal K115, K119, K120, K122, K125 and K128 were all highly modified, with acetylation and methylation species observed on these lysine residues. These H2A C-terminal lysines are important in DNA binding, where the basic side-chains interact with the negatively charged DNA phosphate backbone (63). We observed four phosphorylation marks on the C-terminal tail of H2A at residues S105, S112, S123 and S132. *T. brucei* H2A S123 corresponds to *S. cerevisiae* S121 and *S. pombe* S122, which have been shown to be involved in a range of functions such as the DNA damage response, telomere silencing, and chromosomal stability (64–66).

We mapped seven PTMs on the tails of the histone H2A.Z variant (Figure 1B). Tri-methylation was detected at H2A.Z lysine K35 and K43, while the H2A.Z residues K31, K35 and K59 were acetylated. On the C-terminal tail, lysine K164 and K168 were acetylated.

### Modifications of *T. brucei* histone H2B isoforms

The *T. brucei* histone H2B isoforms are the most divergent of the four core trypanosomal histones (67). On H2B, we detected 12 PTMs of which four were novel (Figure 1C). H2B lysines were mono-methylated at K4, K9, K12, K15 and K48, with acetylation observed at K48, while arginine 11 was mono-methylated. We detected hydroxylation of tyrosine on H2B at Y28, a conserved modification also seen in *T. cruzi*, and a possible analogue to hydroxylated tyrosine 39 on H2B in humans (61). *T. brucei* H2B only shares ∼48% identity with other eukaryotic H2Bs and the N-terminal tail appears truncated when compared to that of *S. cerevisiae*. Investigation of H2B phosphorylation in budding yeast, fruit flies, mice, and humans has uncovered an association with a wide range of functions, including DNA fragmentation, double-stranded breaks, activation of stress-response genes, as well as apoptosis, all of which have been linked to phosphorylation of H2B polar amino acids (57,68–70). *T. brucei* H2B appears to be similarly modified, with phosphorylation observed on threonine (T2, T6, T24) and serine (S38) residues. Two of these H2B PTMs are conserved in trypanosomes, with phosphorylated T2 and T24 observed in *T. cruzi* (71,72). *T. brucei* H2B T24 aligns with phosphorylated *D. melanogaster* H2B S33, which is mediated by the carboxyl-terminal kinase of TAF1 and is involved in transcriptional activation (56,57,73). Four phosphorylation modifications were observed on peptides that include S35, S38, S41, T43, T46 and S52 residues in the globular domain of H2B. However, incomplete fragmentation of these peptides did not allow unambiguous placement of these modifications.

On histone H2B.V, we detected 16 modifications of which three have not been described previously (Figure 1D). As observed with H2A.Z, H2B.V appears less modified compared to the canonical counterpart in later-branched eukaryotes, in agreement with observations in *T. cruzi* (34,71,72). Novel PTMs identified include H2B.V P1ac, R8me2 and K17me1.

### Modifications of *T. brucei* histone H3 isoforms

On histone H3, we identified 50 PTMs of which 26 have not been reported before (Figure 1E). We observed mono- or di-methylation on multiple H3 N-terminal and globular arginine residues at residues R2, R8, R36, R37, R39, R46, R49 and R66. *T. brucei* H3 R2 and R8 align with conserved R2 and R8 residues on human H3, which have been linked to gene expression and transcriptional repression in their methylated forms, respectively (56). All-atom molecular dynamics simulations of the nucleosome core particle (NCP) found that a region of histone H3 (residues H39 to R49) acts as a ‘latch’ by holding the nucleosomal DNA ends attached to the underlying DNA gyre (74). Disruption of the interactions between the H3 latch and the DNA end lead to DNA breathing, unwrapping, or sliding. The H3 latch region is conserved across the eukaryotic lineage (see histone H3 alignments in Supplementary Table S5, sheet 1). Three arginine latch residues (R40, R42 and R49) stabilize the NCP by interacting with the backbone or minor groove of the DNA molecule (74). These arginines are conserved in *T. brucei* (R37, R49 and R46), all of which were found to be mono-methylated. Methylation of these residues may cause destabilization of the NCP, as methylation of R42 (R39 in *T. brucei*) is predicted to cause DNA unwrapping at the DNA entry site to the nucleosome, leading to DNA ‘breathing’ (75). In *H. sapiens*, mono-methylation of H3 K56 is mediated by the G9a methytransferase and is associated with DNA replication (76). This residue aligns with *T. brucei* R53, a conservative amino acid substitution, which was found to be mono-methylated.

H3 lysine K4 was mono-, di, or tri-methylated as previously observed (25). Tri-methylated H3 K4 marks canonical Pol II TSSs in *T. brucei* and was found to be enriched on H2A.Z:H2B.V containing nucleosomes (77). This histone mark appears to be conserved on eukaryotic H3 histones and is a known mark for transcriptional activation in humans (56). We observed H3 N-terminal lysine residues K10, K11, K16, K17, K19, K20 and K23 in mono-, di-, tri-methylated and acetylated states.

The *T. brucei* H3 N-terminal tail diverges sharply from that of later-branched eukaryotes, sharing only ∼32% identity and lacks the near-universally conserved H3K9 which is associated with contrasting functions depending on its modification state. In its tri-methylated state, it is involved in transcriptional repression via heterochromatin formation mediated by the chromatin-modifying enzyme heterochromatin protein 1 (HP1) (78). Conversely, acetylated H3 K9 promotes transcriptional activation mediated by the acetyltransferases Gcn5 and SAGA (79). It is unclear whether *T. brucei* H3 K10 or K11 is a possible functional equivalent of H3 K9 in later-branched eukaryotes, as the surrounding amino acid sequence is notably different, suggesting interaction with structurally distinct protein surfaces. An interesting finding is that these residues were only observed to be acetylated in BF cells, which may indicate their possible involvement in life-cycle specific epigenetic functions.

*T. brucei* H3 K19 aligns with *S. cerevisiae* and *H. sapiens* H3 K23ac, a known mark of transcriptional activation mediated by SAGA and CBP chromatin remodellers (79,80). *T. brucei* H3 K23 aligns with H3 K27 in budding yeast and fruit fly where it has been linked to various functions depending on the modification status. These include enhancer function and gene expression when acetylated, and Polycomb repression and X-chromosome inactivation when methylated (56,81,82).

At *T. brucei* H3 K32 all three methylation states were observed. This H3 residue aligns with budding yeast and mouse K36 where it is involved in SETD2 mediated transcriptional activation when methylated, and SET2 mediated gene repression when di-methylated in *S. cerevisiae* (56,83–86). In the H3 globular domain, we found K61 to be mono-, di- or tri-methylated or acetylated. *T. brucei* H3 K61 aligns with H3 K64 in mouse and humans where it is involved in transcription and nucleosome dynamics when acetylated, and pericentric heterochromatin formation when tri-methylated (56,87,88). Phosphorylated serine was observed on *T. brucei* H3 at S25 and S29, a possible analogue for the mammalian histone H3.3 mitosis-specific phosphorylated S31 (56).

We observed five PTMs on histone H3.V, of which two (acetylated A1 and mono-methylated K4) mapped to the N-terminal tail (Figure 1F). In the globular domain, lysine K82 was found to be tri-methylated or acetylated.

### Modifications of *T. brucei* histone H4 isoforms

Trypanosomal H4 is the most conserved of the core histones. In this study, we mapped 45 PTMs to H4, of which 18 are novel (Figure 1G). The H4 N-terminal lysine residues interact with the acidic patch formed by the H2A-H2B dimer on adjacent nucleosomes and are key mediators of inter-nucleosome interactions and formation of higher-order chromatin (59). We observed that *T. brucei* N-terminal H4 lysine residues K2, K4, K5, K10, K14, K17 and K18 were mono-, di-, tri-methylated or acetylated. Acetylation of the N-terminal lysine residues would disrupt the formation of salt-bridges between H4 and the acidic patch, leading to chromatin decondensation (89). It has recently been demonstrated that depletion of HAT2 in *T. brucei* by RNAi led to a decrease in H4 N-terminal lysine acetylation at Pol II TSSs, a concomitant reduction of H2A.Z deposition, and shifts the site of transcription initiation upstream (25). Four of these *T. brucei* H4 N-terminal lysines align with conserved H4 residues in later-branched eukaryotes that have defined functions. In humans H4 K5 acetylation, mediated by HAT1, is involved in histone deposition, and in *S. cerevisiae* is important in cell cycle progression (56). *T. brucei* H4 K4, the likely equivalent of H4 K5 in later-branched eukaryotes, is acetylated by HAT3 and appears to be acetylated upon nuclear import by a MYST-type acetyltransferase (90). Trypanosomal H4 K10 aligns with *S. cerevisiae* and *H. sapiens* H4 K12, where it is involved in histone deposition and stress response when acetylated (SET5 mediated) or mono-methylated (HAT1 mediated), respectively (91,92). In *T. brucei*, acetylated H4 K10 was found to localize to putative Pol II TSSs (21).

H4 K14 in trypanosomes aligns with H4 K16 in later-branched eukaryotes, where it functions as a sequence-specific transcription factor binding site in mouse and human (56). *T. brucei* H4 K18 corresponds to K20 in human and *Drosophila* H4, which are methylation targets associated with SETD8 mediated transcriptional silencing and Pr-SET7 mediated mitotic condensation (93,94). We observed H4 C-terminal lysine K93 in mono- or di-methylated states.

Methylation of several arginine residues was observed on the H4 N-terminal tail as well as in the globular domain. Arginine R21, R25 and R89 were mono- or di-methylated, while R15, R29, R34 and R91 were mono-methylated. *T. brucei* H4 R21 and R33 align with mouse H4 R23 and human R35, respectively, known sites of arginine methylation (61,95). Three hydroxylated tyrosine residues were detected on H4; one in the globular domain at Y49 (analogous to human Y51 found to be similarly modified), and two on the C-terminal tail at Y96 and Y98 (61). These hydroxylation marks are conserved in trypanosomes and were also observed on *T. cruzi* histone H4 (71).

Histone H4 variants are remarkably rare in eukaryotes with only a few identified in diverse species like *T. brucei*, soybeans, wheat, fungi, ciliates, and recently humans (96,97). On H4.V, we identified 20 modifications of which nine are novel (Figure 1H). We detected mono- or di-methylation on H4.V K10, while lysines K14, K17 and K18 were mono-, di-, or tri-methylated, as previously reported (25). We mapped mono-methylation marks to the H4.V N-terminal tail at R15, and to globular region at R21, R25, R29, R33 and R37. On the H4.V C-terminal tail, lysine K93 was mono- or di-methylated. As observed on canonical H4, we found three hydroxylated tyrosine residues on H4.V. Of these three, only Y49 could be mapped to a specific residue. Although the C-terminal tail peptides displayed hydroxylation modifications, fragmentation did not allow unambiguous localisation to Y96 or Y98. However, taking into account the conserved nature of the *T. brucei* H4 histone isoforms, it is possible that both H4.V Y96 and Y98 residues may be hydroxylated similar to that observed on canonical H4.

### *T. brucei* core histones are decorated by various combinatorial modification patterns

Individual histone PTMs do not always correspond to a single functional state. Instead, PTMs act in a combinatorial manner to establish intricate patterns that regulate genomic functions and other chromatin mediated mechanisms through creation of a specific binding surface or disruption of existing interactions (22,23). The middle-down MS approach used in this study allows the detection of complex combinatorial PTM patterns on endoproteinase digested histone peptides (37). This technique has been successfully applied to map *cis*-histone cPTMs in a wide range of organisms (38,72,98). Using Histone Coder (37) to filter and analyze the MS/MS data and unambiguously assign PTMs to specific residues, we identified 855 cPTMs, including 110 cPTMs observed in three or more replicates. A comprehensive list of observed cPTMs is provided in Supplementary Table S9, sheet 4. It was estimated that 76–100% of the N-terminal peptides of H2A, H2B, H3 and H4 were modified. Relative levels of modified vs. unmodified peptides are shown for the major histone isotypes in sheet 1 of Supplementary Tables S1, S3, S5, and S8.

We found that histone H2A is extensively modified with cPTMs. The majority of cPTM patterns we observed localized to the H2A N- and C-terminal tails, although some were observed on the globular domains. We identified a total of 89 cPTMs on H2A, of which 47 were present as binary marks. The H2A N-terminal tail was mainly decorated with binary PTMs which largely consisted of a combination of mono- or di-methylated K/R, and phosphorylated T/S residues. We observed that mono-methylated K4, K8 and K12 were the most frequently observed modifications present in H2A N-terminal cPTMs. In combination with phosphorylated T2, these formed the most frequently observed binary marks identified on the H2A N-terminal tail. Although we detected H2A K4me3 and K8me3 as singular PTMs on H2A N-terminal peptides, these modifications were not observed to form any cPTMs and may, therefore, function individually.

cPTMs were not evenly distributed between the two tail regions of H2A, with 71 (79.7%) of the cPTMs localizing to the C-terminal tail. Acetylated K119, K120 and K122 were the most frequently observed cPTM forming residues bearing these modifications.

The C-terminal H2A tail contains six lysine residues that were present in multiple hyperacetylated states, with up to five acetylated lysines coexisting on the same peptide. Lysine residues K115, K119, K120, K122 and K125 bore the bulk of acetylation marks found to form cPTM patterns, with K119ac and K120ac observed most frequently in hyperacetylated H2A peptides. These lysine residues are exposed to the nucleosomal surface and are important for DNA interaction (8,63). Hyperacetylation of these C-terminal tail residues would negate the positive charge of the lysine side-chains and disrupt histone-DNA interactions and nucleosomal stability, probably producing a more relaxed, euchromatic state amenable to transcription.

**Figure 2.**
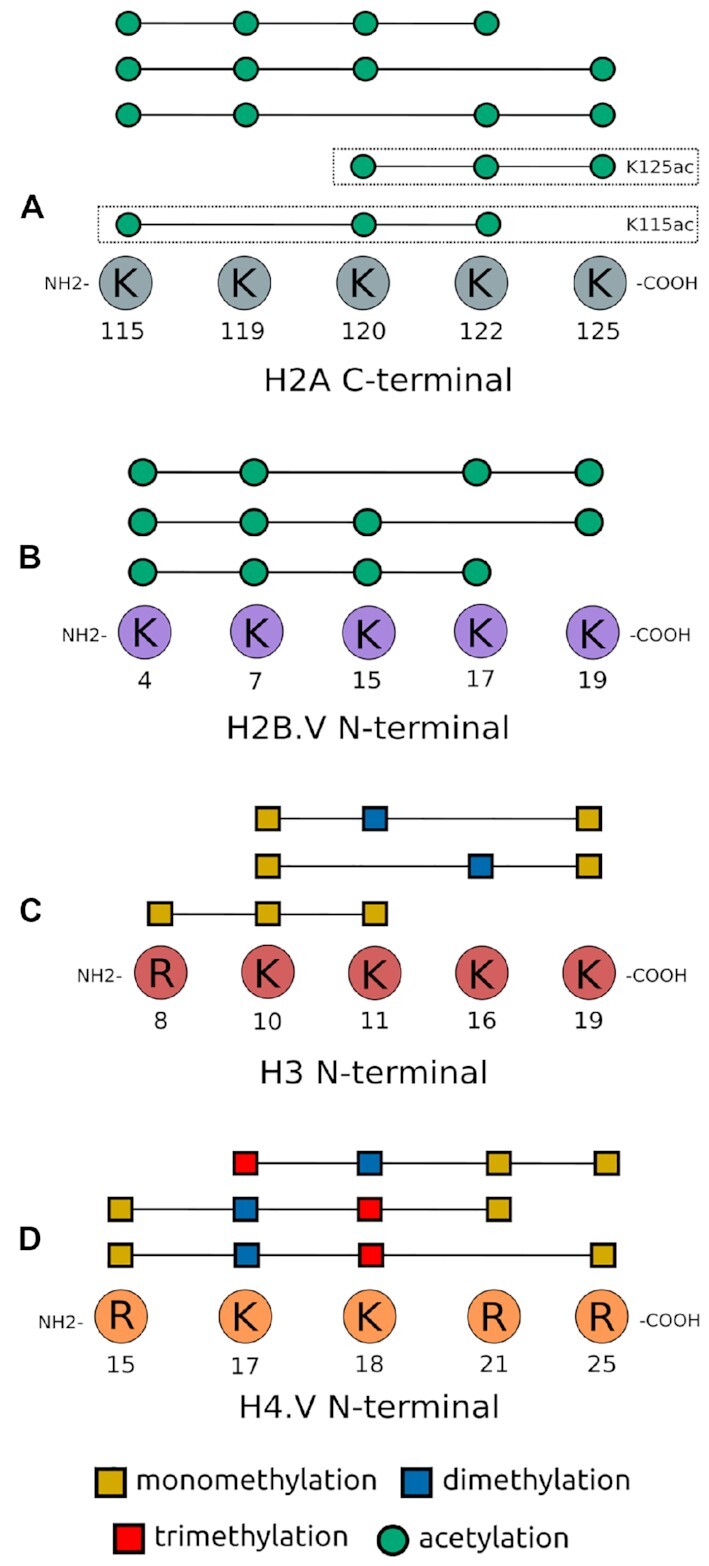
Combinatorial PTM (cPTM) patterns observed on histone tails. Schematic of the three most frequently observed cPTM patterns seen on histone tails, where each cPTM consists of three or four individual PTMs. Individual cPTM patterns are indicated with each cPTM pattern represented by individual lines. (**A**) C-terminal H2A (also showing H2AK115ac and H2AK125ac hyperacetylation patterns discussed later on), (**B**) N-terminal H2B.V, (**C**) N-terminal H3 and (**D**) N-terminal H4.V. Supplementary Table S9, sheet 4 provides a list of cPTMs observed on all core histones.

Figure 2A shows the top three hyperacetylated cPTM patterns observed on C-terminal H2A lysine residues, with each cPTM consisting of four individual acetylation marks: K115acK119acK120acK122ac, K115acK119acK120acK125ac and K115acK119acK122acK125ac. Hyperacetylation of the H2A C-terminal tail to this extent has not been observed in other eukaryotes, and appears to be trypanosome specific. Similar acetylation patterns have been observed on conserved lysine residues in *T. cruzi* H2A (71). This suggests that H2A C-terminal hyperacetylation patterns could have been established before trypanosomal speciation, and may serve distinct functions in these organisms.

We found that the N-terminus of H2A.Z was hyperacetylated which is comparable to what is seen in *S. cerevisiae* and *T. cruzi* (72,99). It has been demonstrated that acetylated H2A.Z localizes to putative Pol II transcription start regions in *T. brucei* (25). Knock-down of the histone acetyltransferases HAT1 and HAT2 by RNAi decreased the level of acetylation of H2A.Z N-terminal lysine residues, leading to a loss of H2A.Z deposition at Pol II TSS, a 50% reduction of total mRNA levels, and shifts of the site of transcription initiation upstream (25).

We observed that combinatorial PTMs identified on histone H2B were localized at the N-terminus and extended into the α-1 histone fold. cPTMs spanning up to 34 residues were observed and consisted of mono-methylated K4 in combination with phosphorylated serine 24 or threonine 38. The cPTMs that we identified on histone H2B.V localized to the N-terminal tail, which was found to be hyperacetylated, with up to four acetylation marks coexisting on the same peptide. Figure 2B shows three hyperacetylated cPTMs observed on N-terminal H2B.V peptides: K4acK7acK17acK19ac K4acK7acK15acK19ac and K4acK7acK15acK17ac. Similar patterns of hyperacetylation have been identified in *T. cruzi*, suggesting a conserved function in trypanosomes (71). The N-terminal tail of the H2B.V variant is elongated compared to canonical H2B, with the hyperacetylated lysine residues observed on H2B.V absent from the H2B N-terminal tail. The H2B tail protrudes from between the DNA gyres on the nucleosome disk opposite to the side of DNA entry and exit, where these basic residues interact with DNA, and are associated with gene-specific transcriptional activation when acetylated (8,100). *T. brucei* H2B.V dimerizes exclusively with H2A.Z and is enriched at regions where Pol II transcription starts (21,101). Although the mode of action is unknown, H2A.Z-H2B.V containing chromatin is less stable and may be resistant to chromatin condensation, thereby producing a euchromatic state primed for transcription (102). The observed level of N-terminal lysine hyperacetylation on H2B.V would disrupt histone-DNA interactions, and may contribute to euchromatin formation.

Most of the cPTMs observed on H3 localized to the N-terminus, although some cPTMs were observed in the H3 globular domains. The majority of cPTMs identified were methylated to variable degrees, with binary marks consisting of a combination of arginine and lysine methylation states. Mono-methylated K10, and tri-methylated K16 and K17 were the most frequently observed pattern forming PTMs bearing these marks. Interestingly, we did not find that K4me3, K16ac, K23ac, K32me3, R49me1, R53me1 and K61me1 were present in cPTMs, but existed as individual marks. We found combinatorial PTMs containing three individual methylation modifications on the N-terminal tail of H3. Figure 2C shows K10me1K11me2K19me1, K10me1K16me2K19me1 and R8me1K10me1K11me1 cPTMs. The nine lysine residues present on the H3 N-terminal tail all formed part of cPTMs, with two acetylation and multiple methylation modifications coexisting. However, no hyperacetylated H3 peptides were detected. Intriguingly, *T. brucei* H3 displays a much smaller combinatorial PTM repertoire, in contrast to most other eukaryotes where H3 is the most densely modified core histone and exhibits a large number of complex cPTM patterns (36,38).

The bulk of cPTMs that we identified on histone H4 localized to the N-terminal tail, with only a few extending into the α-1 histone fold domain. The H4 tail appears to be hypermodified, with up to five modifications coexisting on the same peptide. The N-terminal tail contains two arginine and seven lysine residues, all of which were observed to form part of cPTM patterns. The H4 N-terminal tail protrudes from the side of the nucleosomal disk where the basic lysine residues are important for inter-nucleosomal binding. Acetylation of these lysines disrupts inter-nucleosomal interactions, causing chromatin destabilization that give rise to a more relaxed chromatin state amenable to transcription (89,103). Hyperacetylated H4 peptides, containing up to five acetylated lysine residues, have been observed in a wide range of eukaryotes such as *S. cerevisiae*, *D. melanogaster* and the related *T. cruzi* (72,99,104). In *T. brucei*, the H4 tail does not appear to be extensively acetylated, with only three acetylated lysine residues observed to co-occur on the same peptide. Lysine residues K2, K4, K5, K10, K14, K17 and K18 present in cPTMs were seen in acetylated, mono-, di- and tri-methylated states. Of the seven N-terminal acetylated lysines, we observed K2, K17 and K18 were most frequently present in cPTM patterns. Acetylated A1 was the only N-terminal PTM not observed to form cPTM patterns. With the exception of methylated R25, R29 and R89 no cPTM patterns were observed on the globular regions of H4.

Although the *T. brucei* H4 isoforms are highly similar and share 91 conserved residues, dissimilar cPTM patterns were observed on H4.V compared to those seen on canonical H4. Unlike H4, cPTMs on H4.V extended much further into the central histone fold domain. The patterns that we observed consisted of lysine and arginine residues methylated to various degrees, with up to five modifications coexisting on the same peptide. No acetylated N-terminal cPTMs were detected on H4.V. This is perhaps not surprising as this histone variant is associated with regions of transcription termination, and H4.V N-terminal lysine acetylation may negate the epigenetic function of H4.V at regions of Pol II transcription termination. Modified K17 and K18 residues were present most frequently in cPTMs. Figure 2D shows the three most frequently observed H4.V cPTMs, each consisting of four individual modifications: K17me3K18me2R21me1R25me1, R15me1K17me2K18me3R21me1 and R15me1K17me2K18me3R25me1. Histone hypermethylation, like the cPTMs described above with three or more methylated K or R residues occurring on the same peptide, is generally associated with transcriptional repression, which is consistent with the localization and function of H4.V at regions of transcriptional termination in *T. brucei* (105).

### C-terminally hyperacetylated H2A marks Pol II transcription start regions

With the exception of the SL RNA genes, *T. brucei* lacks clearly detectable Pol II promoter and regulatory DNA sequences. Instead, it deposits histone variants and PTMs to demarcate Pol II transcription start and termination boundaries at the polycistronic gene arrays (21). Specifically, H2A.Z and H2B.V co-localize with H3K4me3 and H4K10ac at regions of Pol II transcription initiation. In later-branched eukaryotes, H2A.Z is associated with nucleosomes that border the nucleosome depleted region, which overlaps with the Pol II transcription start site (106). In a previous study, we have shown that a clear nucleosome depleted region is absent in *T. brucei*, but that a region of lower nucleosome density is present upstream of the first gene in a Pol II transcribed PTU (107).

The degree of hyperacetylation observed on the trypanosomal H2A C-terminal tail lysines could lead to chromatin decondensation, as acetylation would negate the positive charge of the basic lysine residues important in DNA binding (63). This reduction in DNA binding could have a similar effect to that of the mammalian Barr body-deficient histone variant H2A.Bbd which lacks the docking domain involved in H1 and H4 binding, and associates with transcriptionally active chromatin (63,108). Incorporation of H2A.Bbd into chromatin causes structural destabilization of the nucleosome and abolishes the ability of H2A.Bbd containing nucleosomes to condense into higher order chromatin (109). It has been established that an open, euchromatic state allows the Pol II transcriptional complex to more easily access DNA and results in fortuitous transcription initiation in *T. brucei* (110). Indeed, BF trypanosomes have more heterochromatic chromatin than PF cells which presumably contributes to transcriptional silencing of silent VSG expression sites in the BF life stage of the parasite (111).

In order to investigate the genomic distribution and potential functional involvement in transcription of hyperacetylated H2A via MNase-ChIP-seq, we raised polyclonal antibodies in New Zealand rabbits using differentially acetylated synthetic peptides. The two cPTMs selected were observed in both PF and BF trypanosomes, and contained three acetylated lysine residues: K115acK120acK122ac and K120acK122acK125ac, henceforth referred to as H2AK115ac and H2AK125ac, respectively. These marks were chosen as targets as they were observed at similar frequency in BF and PF samples, and this level of hyperacetylation would disrupt histone-DNA binding (63,109). Antibodies were affinity-purified and potential cross-reactivity was evaluated via indirect ELISA against modified and unmodified H2A C-terminal tail peptides. ELISA, peptide-based competition assays, and dot-blot analysis demonstrate the specificity of the antibodies used (Supplementary Table S9 sheet 1, and Supplementary Figure S2). Of critical importance was our observation that the H2AK115ac and H2AK125ac antibodies did not cross-react. We used these cPTMs to investigate whether H2A hyperacetylation in any combination served interchangeable epigenetic functions. In addition we investigated if cPTMs in specific arrangements localized at distinct genomic regions, implying differential functionality. Anti-histone H3 was used as a ChIP control. We do not preclude the possibility that nearby PTMs may bias the analysis to histones only containing the marks defined by the synthetic peptides used to raise the antibodies.

A native MNase-ChIP-seq technique was used as described previously (44). The resulting DNA fragments were aligned to the *T. brucei* 427 reference genome, and peaks of enrichment were identified. Visual inspection of nucleosomal profiles of input and ChIP DNA revealed no striking difference between BF and PF life-cycle stages. This is in agreement with previous findings where genome-wide nucleosomal architecture between both *T. brucei* life cycles was found to be remarkably similar (107). Intersection of ChIP-seq reads revealed that H2AK125ac and H2AK115ac displayed dissimilar genomic distributions (Supplementary Figure S3), indicating that differential hyperacetylation of the H2A C-terminal lysine residues could have different epigenetic functions. We did not observe enrichment of either cPTM at Pol I or Pol III transcribed genes, or at genes which are transcribed in a life-cycle specific fashion (procyclin, PAG, and VSG genes, or genes at BESs). H2AK115ac were found to be distributed within and between PTUs, on coding and intergenic regions, and were not correlated with classes of genes that were relevant to a specific life cycle stage. Even though BF and PF H2AK115ac reads, alignment rates, and mean number of reads mapped per base pair is comparable, MACS2 identified three times more peaks in the BF (523 peaks) versus the PF sample (155 peaks). This may indicate that, although this cPTM is present in both life stages of the parasite, this pattern may be more essential to chromatin processes in bloodstream form than procyclic form cells.

**Figure 3.**
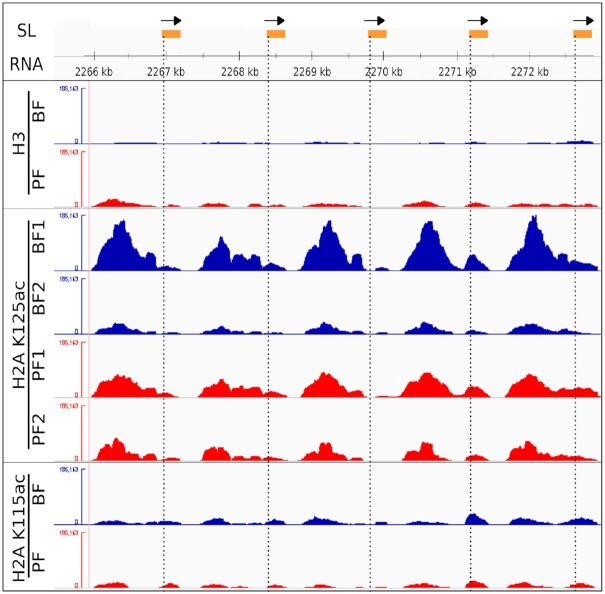
ChIP-seq profiles at spliced leader (SL) RNA genes. Schematic of individual SL RNA transcription units (250 bp) that include the bipartite upstream sequence and initiator elements as well as the SL RNA transcript. SL RNA genes are indicated in orange with the direction of transcription indicated with black arrows. Nucleosomal profiles of immunoprecipitated samples at individual SL RNA Pol II transcription units on chromosome 9 are shown for BF and PF histone H3, BF and PF histone H2AK125ac replicates, and BF and PF histone H2AK115ac. BF samples are indicated in blue, while PF samples are indicated in red.

However, we found that H2AK125ac was highly enriched at Pol II transcribed SL RNA loci. We observed peaks of enrichment (where ChIP DNA signal is five fold higher than background) over the entire ∼42.8 kb length of the SL RNA array (Figure 3). The presence of hyperacetylated H2A at loci where Pol II transcription occurs at high rates suggests an intriguing correlation between deposition of the H2AK125ac cPTM and Pol-II-mediated transcription. The repeated series of peaks of ChIP signal seen extending over the entire SL RNA array could be a consequence of the repetitive nature of the SL RNA gene array, where H2AK125ac is present upstream of the individual SL RNA genes. No significant enrichment of H3 or H2AK115ac was observed on the SL RNA array itself.

**Figure 4.**
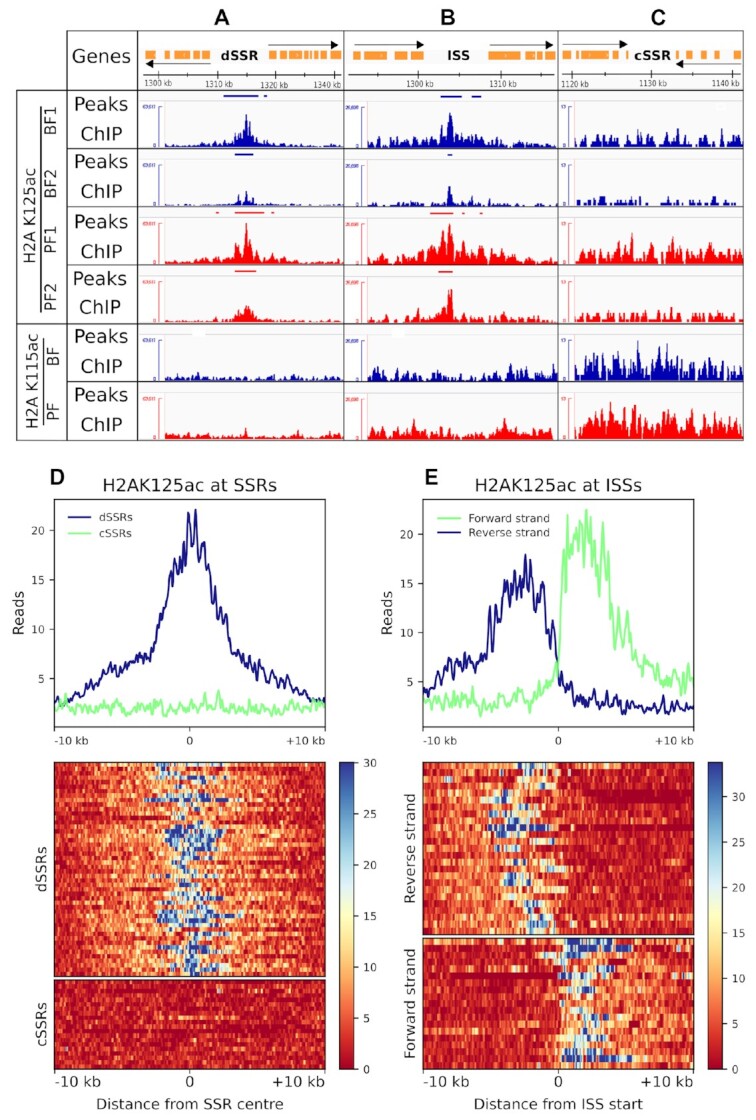
Distribution of hyperacetylated H2A cPTMs across different configurations of polycistronic Pol II transcription units. The open reading frames are shown in the top track with orange boxes, with the direction of Pol II transcription indicated by black arrows. Peaks of enrichment and of ChIP profiles for BF (blue) and PF (red) are shown at (**A**) a divergent strand switching region (dSSR) (chromosome 7: 1302589–1326571) where Pol II transcription initiates bi-directionally, (**B**) an internal Pol II start site (ISS) between two head-to-tail transcription units (chromosome 9: 820027–848077), and (**C**) a convergent strand switch region (cSSR) (chromosome 7: 1118676–1141170) where Pol II transcription terminates. H2AK125ac read enrichment plots and heatmaps for 10 kb regions up- and downstream relative to (**D**) the center of SSRs and (**E**) beginning of ISSs are shown.

At the polycistronic gene arrays, we found that the profiles of hyperacetylated H2A ChIP samples displayed a striking enrichment of H2AK125ac peaks at dSSRs, with H2AK125ac containing nucleosomes observed along the entire dSSR in both BF and PF samples (Figure 4A and Supplementary Figure S4A). The observed H2AK125ac enrichment is centered at the midpoint of the dSSRs and does not significantly extend further downstream into the PTU (Figure 4A and D, and Supplementary Figure S5A–D). We observed that divergent SSRs on all 11 megabase chromosomes were highly enriched in H2AK125ac, with all of the 74 dSSRs examined displaying enrichment of H2AK125ac peaks in both BF and PF trypanosomes across both replicates.

H2AK125ac peaks were also found to be present at regions where Pol II transcription terminates and then reinitiates at internal start regions (ISS) (Figure 4B and Supplementary Figure S4B). Internal regions between gene clusters arranged head-to-tail where transcription stops and then starts may be affected by the presence of an actively transcribed rRNA or tRNA gene. These could provide a kinetic block to the Pol II transcriptional complex, and contribute to its termination. Of the 62 ISSs we investigated, 95% (*n* = 59) were enriched with H2AK125ac containing nucleosomes. Unlike what was observed at dSSRs, H2AK125ac containing nucleosomes localized towards the 5′ side of these head-to-tail regions, and extended with a diminishing gradient into the Pol II PTU (Figure 4E and Supplementary Figure S5E–H).

Histone variants H3.V and H4.V localize to transcription termination regions in convergent SSRs where they regulate Pol II transcription termination, possibly through formation of heterochromatic regions (21,112,113). We found that hyperacetylated H2AK125ac containing nucleosomes are depleted from cSSRs, which would be in agreement with H2AK125ac chromatin being more euchromatic. The presence of H2AK125ac at these regions could possibly negate the heterochromatic structural effects caused by the H3.V and H4.V variants and lead to improper transcription termination or transcriptional read-through (Figure 4C and D, and Supplementary Figure S4 C and S5A–D).

Supplementary Figure S6 shows the striking localization of H2AK125ac at Pol II transcription initiation regions across chromosome 11. Although no significant H2AK125ac enrichment is found outside of the Pol II transcription initiation regions, H2AK125ac containing nucleosomes are not entirely absent from the Pol II PTUs. Low levels of H2AK125ac are observed along the entire PTU, where it may contribute to the maintenance of an open chromatin structure that is amenable to constitutive Pol II transcription. We did not observe enrichment of H2AK115ac marks at dSSRS, head-to-tail transcription units, ISSs or cSSRs, indicating that in *T. brucei* specific patterns of H2A C-terminal hyperacetylation are used for distinct functions.

Histone H2A.Z and the Pol II sub-unit RPB9 are useful markers for Pol II transcription initiation regions in trypanosomes, together with epigenetic marks like H3K4me3 and H4K10ac (77,114,115). It is believed that heterotypic H2A.Z:H2A containing nucleosomes occur at low levels in *T. brucei* (21,25,101). To investigate the genomic relationship of RPB9, H2A.Z and H2AK125ac, RPB9 and H2A.Z ChIP-seq data from a previous study were retrieved and processed with the same workflow pipeline used in this study (115).

**Figure 5.**
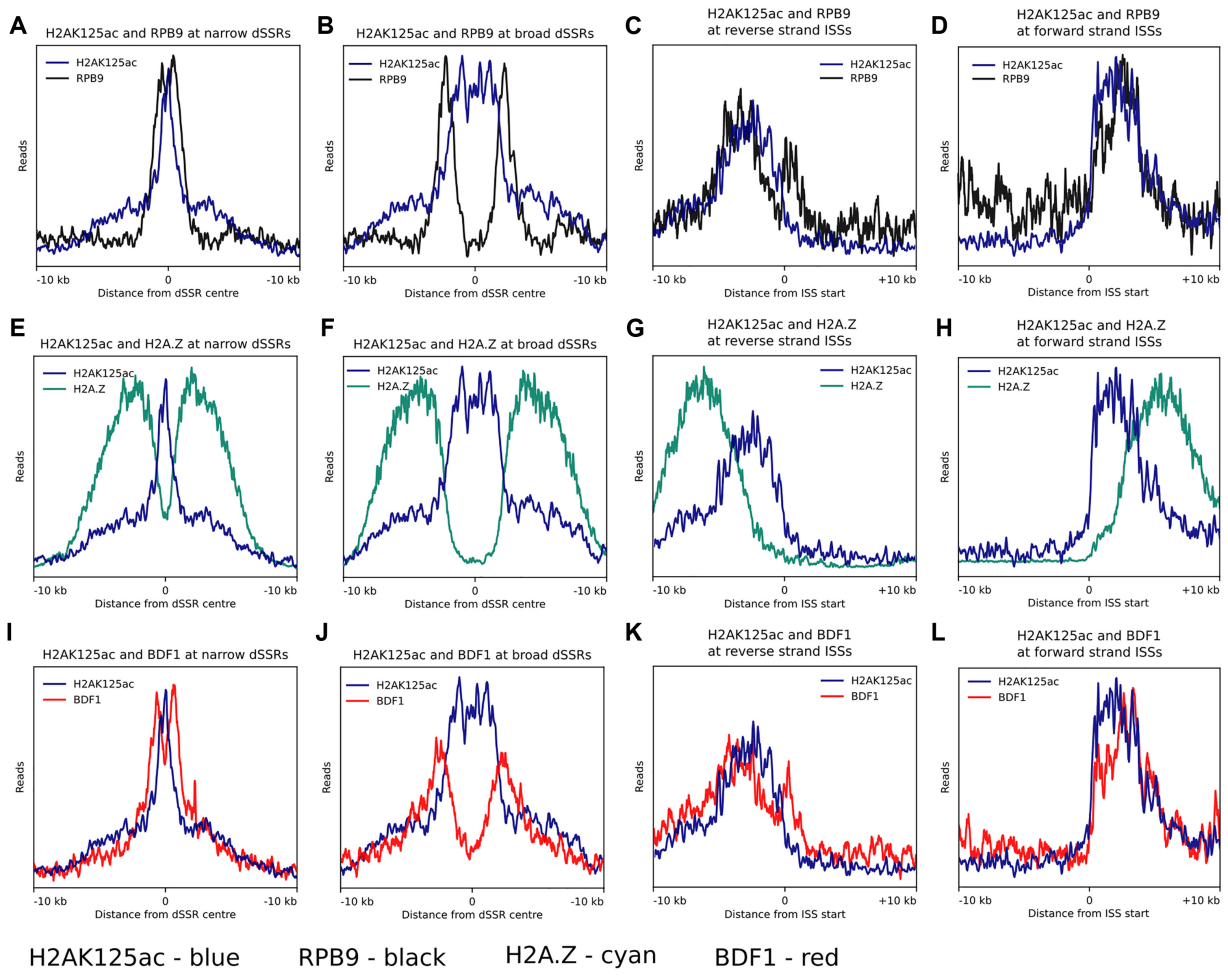
H2AK125ac, RPB9, H2A.Z, and BDF I distribution at different Pol II transcription start regions. Superimposition of read enrichment profiles around a 20 kb region (10 kb up- and downstream) of (**A**) H2AK125ac (blue) and RPB9 (black) at narrow dSSRs, (**B**) H2AK125ac and RPB9 at broad dSSRs, (**C**) H2AK125ac and RPB9 at reverse strand ISSs, (**D**) H2AK125ac and RPB9 at forward strand ISSs. (**E**) H2AK125ac and H2A.Z (cyan) at narrow dSSRs, (**F**) H2AK125ac and H2A.Z at broad dSSRs, (**G**) H2AK125ac and H2A.Z at reverse strand ISSs, (**H**) H2AK125ac and H2A.Z at forward strand ISSs. (**I**) H2AK125ac and BDF1 (red) at narrow dSSRs, (**J**) H2AK125ac and BDF1 at broad dSSRs, (**K**) H2AK125ac and BDF1 at reverse strand ISSs, (**L**) H2AK125ac and BDF1 at forward strand ISSs.

Visual inspection of RPB9 enrichment showed twin peaks that flank dSSRs, as previously reported (115), and are present immediately downstream of the dSSRs (Supplementary Figure S7A). Initial read enrichment profiles of RPB9 displayed a single broad peak at dSSRs (Supplementary Figure S8A), caused by the stacking of heatmap values over these regions. However, the heatmap traces indicated that RPB9 was enriched at bordering loci that correlate with the width of the individual dSSR. Sorting of dSSRs based on their width into ‘narrow’ (≤4000 bp) and ‘broad’ (>4000 bp) distributions indicate that RPB9 does indeed form two peaks, with a furrow present between the two peaks (Supplementary Figure S8B). Applying the same sorting to H2AK125ac peaks to compare it to the distribution of RPB9, we found that hyperacetylated H2AK125ac containing nucleosomes are flanked by RPB9 peaks at broad and narrow dSSRs (Figure 5A and B, respectively, and Supplementary Figure S7A). Supplementary Figure S9 shows heatmap analysis of BF and PF H2AK125ac sorted into narrow and broad dSSRs, where H2AK125ac can be observed to be enriched across the entire dSSR. At regions where transcription terminates and then reinitiates, RPB9 forms a single peak slightly downstream of the internal start site (Supplementary Figure S7B and S8C). At these loci, both RPB9 and H2AK125ac peaks co-localize to the start of these regions where they appear to overlap (Figure 5C and D).

H2A.Z containing nucleosomes formed twin peaks that flank dSSRs, present downstream of the dSSRs (Supplementary Figures S7A and S10A). H2AK125ac containing nucleosomes are present between the two H2A.Z peaks at both broad and narrow dSSRs, with traces of H2AK125ac sharply diminishing where H2A.Z increases (Figure 5E and F). At internal start regions, H2A.Z forms a single peak downstream of the internal start site (Supplementary Figure S7B). At these sites, H2AK125ac peaks localize to the beginning of the internal start site, towards the 5′ side of the single H2A.Z peak, with H2A.Z containing nucleosomes enriched further downstream over a broader area (Figure 5G and H, Supplementary Figures S7B and S10C).

Bromodomain-containing proteins are known to bind acetylated histones (116). To gain functional insight into the potential interaction between chromatin-binding proteins and hyperacetylated H2AK125ac, ChIP-seq data of six bromodomain factors (BDFs 1–6) were retrieved and processed with the same workflow pipeline used in this study (26).

Class I BDFs (which include BDF1, BDF3 and BDF4) were previously shown to form sharp peaks at Pol II transcription start regions (26). Heatmap analysis of these class I BDFs showed sharp peaks at dSSRs when sorted into narrow and broad dSSRs (Supplementary Figure S11A–C), with twin peaks appearing on either side of the dSSR center, indicating that this arrangement is sensitive to the direction of transcription. Superimposing BDF1 (as a representative) with BF H2AK125ac indicate that these class I BDFs flank the H2AK125ac peaks at narrow and broad dSSRs, similar to what is seen for RPB9, localizing to regions between H2AK125ac and H2A.Z containing nucleosomes (Figure 5I and J). At ISS, class I BDFs form a single peak immediately downstream of the ISS (Supplementary Figure S11D–F), similar to that of RPB9, where they co-localise with H2AK125ac (Figure 5K and L).

Class II BDFs (which include BDF2, BDF5 and BDF6) were shown to be broadly enriched at transcription start regions, with enrichment patterns similar to that of H2A.Z (26). These BDFs did indeed show distribution profiles similar to that of H2A.Z when sorted into narrow and broad dSSRs (Supplementary Figure S12A–C), indicating that class II BDFs may associate with H2A.Z containing nucleosomes, rather than nucleosomes containing H2AK125ac.

It was previously shown that depletion of the histone acetyltransferase HAT2 by RNAi resulted in a decrease in H2A.Z deposition at TSSs and an increase in the width of Pol II distribution at these regions (25). At these transcription start sites it was found that after HAT2 RNAi, transcription initiation shifted further upstream compared to wild-type cells. It has been hypothesized that sites immediately upstream of canonical TSSs, where H2A.Z localizes, are more accessible than the DNA at the TSS itself (117). These areas of increased DNA accessibility immediately upstream of the TSSs coincide with loci of H2AK125ac enrichment, and may be caused by the observed hyperacetylation of the H2A C-terminal tail.

## DISCUSSION

Trypanosomes have a sophisticated epigenetic system capable of regulating a genome that lacks the complex Pol II transcriptional control machinery found in later-branching eukaryotes. This suggests that many epigenetic features, like employment of histone isotypes and post-translational modifications to regulate genome function, were present in the ancestor to *Excavates* and *Unikonts*. *T. brucei* displays many epigenetic features shared among later-branched eukaryotes. This includes the precise positioning of nucleosomes, utilization of histone PTMs and variants for genome demarcation, and the modulation of heterochromatic gene repression (21,25,107,111,115,118,119). It has become increasingly clear that in *T. brucei* chromatin, and in particular the epigenome, play vital roles in genome regulation and are essential for effective control of genes vital for host immune evasion in the BF mammalian infective life stage (111,120–123).

In this study, the middle-down mass spectrometric analysis of acid extracted core histones of BF and PF trypanosomes revealed an extensive histone epigenetic repertoire of 203 PTMs: 48 on H2A, seven on H2A.Z, 12 on H2B, 16 on H2B.V, 50 on H3, four on H3.V, 45 on H4 and 20 on H4.V. The most comprehensive survey of *T. brucei* histone PTMs published to date (and the first to report H4.V modifications (25)) described 157 PTMs of which 117 were novel. Of these 117 modifications, our data confirms 78 PTMs across all histone isotypes. When including all studies to date, a total of 173 modifications were identified on canonical and variant core histones, of which this study confirms 109, and describes an additional 94 PTMs (Supplementary Table S9, sheet 3). Thus, in total, 267 PTMs have been identified, indicating that *T. brucei* possesses a vast histone PTM repertoire, much larger than expected when considering the comparatively smaller number of histone reader, writers, and erasers present in this organism (67).

The investigation of the methylation status of arginine residues is, to our knowledge, the first time arginine methylation was examined in *T. brucei*. We observed multiple methylated arginine residues present on core histone tails and in their globular domains (Figure 1). Methylation of these residues, catalysed by protein arginine methyltransferases, has been implicated in diverse roles that influence chromatin function, such as promotion or prevention of docking of transcriptional effector molecules, and recruitment of repressive or activating proteins to specific genomic loci (124).

Although some PTMs were observed to occur in isolation on endoproteinase digested histone peptides, a specific PTM does not always correspond to a single functional readout. Instead, a functional histone code can be comprised of numerous individual marks to form complex combinatorial PTM patterns, where each combination gives rise to a different functional readout (24,37). These different cPTM patterns can create specific binding moieties or disrupt existing associations that affect molecular interactions in diverse ways. It has been proposed that combinatorial histone PTMs may also serve as a PTM reservoir, or represent a transient cPTM state (23).

Digestion of histones with unspecific proteases yields short peptide fragments, usually resulting in higher amino acid sequence coverage (25). However, digestion with specific proteases that cut less frequently produce longer peptide fragments that present the advantage of investigating complex co-existing PTM patterns. The middle-down MS technique used in this study was originally optimized for the analysis of long peptides originating from histone tails that are decorated with multiple PTMs. This favours analysis of longer peptides as compared to shorter peptides often originating from the histone core. Nevertheless, our technique allowed the identification of 32 PTMs localising to globular domains that formed part of various cPTM patterns. The majority of cPTMs observed on *T. brucei* core histones localised to the N- or C-terminal tails. These typically extend from the nucleosome core where they participate in intra- and inter-molecular interactions that influence a wide range of functions, such as chromatin stability, higher-order folding, and gene expression (8,23,59,63). These data present the first survey of histone cPTM patterns in *T. brucei*, showing that *T. brucei* histones harbour intricate combinatorial PTMs, some of which appear analogous to those observed in later-branching eukaryotes. Taken together with previous studies of *T. brucei* histone and chromatin epigenetics (21,25,26,107,115), this suggests that a basic histone code was present in the eukaryotic progenitors before trypanosomal speciation, and adds another layer to the epigenetic control used by this organism to regulate chromatin function.

We observed the C-terminal tail of histone H2A to be hyperacetylated with more than 20 distinct combinations observed (Supplementary Table S9, sheet 4). These modification states appear to be specific to trypanosomes and have not been reported in later-branching eukaryotes. The H2A C-terminal lysine residues are important in histone-DNA binding, nucleosome stability, and chromatin condensation (63,109). Hyperacetylation of these H2A lysines could have a structural effect analogous to that of the mammalian H2A.Bbd which lacks the C-terminal lysine residues that are acetylated in *T. brucei* (Supplementary Figure S13). The incorporation of H2A.Bbd into chromatin, found to be enriched at actively transcribed genes, is associated with altered chromatin structure where only 118 bp of DNA is protected from MNase digestion, suggesting that the nucleosome DNA termini are more weakly bound in the H2A.Bbd-containing nucleosome (63,125).

We found that hyperacetylated H2AK125ac is enriched at the Pol II transcribed SL RNA loci and Pol II transcription start regions, where it co-localises with RPB9 upstream of H2A.Z. It has been suggested that DNA upstream of TSS is more accessible than DNA at the TSS itself (117). Reduction of H2A.Z incorporation in nucleosomes at TSSs following HAT2 RNAi causes the site of transcription initiation to shift upstream (25). The H2AK125ac containing chromatin at these upstream loci may be the cause of the observed increased DNA accessibility at these sites. Hyperacetylated H2A containing chromatin could bring about a more relaxed, euchromatic state, where Pol II can more easily access regions of DNA primed for transcription initiation. Another possibility is that Pol II itself recruits specific histone acetyltransferases that deposit acetylation marks on the C-terminal tail of histone H2A to facilitate the actively transcribed chromatin state as observed in *S. cerevisiae* (126). These findings suggest that the euchromatic regions upstream of Pol II transcription start sites may be affected by the deposition of H2AK125ac at these loci. This may allow the Pol II transcriptional machinery to pre-load onto the DNA molecule upstream of transcription start sites, where it is then primed for the trypanosomal equivalent of promoter-firing and promoter escape.

Assessment of genomic distribution of chromatin remodelling factors showed that six bromodomain proteins localise at transcription start regions in *T. brucei* (26). Class I BDFs (BDF1, 3 and 4) were observed to flank H2AK125ac enriched loci at dSSRs, where they localise to the juncture between H2A and H2A.Z containing nucleosomes. At ISSs, the distribution of class I BDFs reflected that of RPB9 and H2AK125ac. Together, these observations indicate that class I BDFs may interact with hyperacetylated H2AK125ac containing nucleosomes. In *M. musculus*, it has been demonstrated that a single bromodomain of Brdt is responsible for selective recognition of diacetylated histone H4 tails which are cooperatively bound and accommodated in a single binding pocket (127). It is possible that in *T. brucei*, hyperacetylation of the H2A C-terminal tail may serve as a binding platform to recruit bromodomain proteins to specific loci to remodel local chromatin structure or recruit other factors necessary for transcription.

As H2AK115ac did not display similar distribution profiles to H2AK125ac, it is possible that the specific arrangement of hyperacetylated H2A C-terminal cPTMs could serve different epigenetic functions. It is possible that genomic regulation by several epigenetic mechanisms on a genome-wide scale in *T. brucei* allows for quicker regulation of genes involved in host adaptation, and contributes a certain ‘robustness’ against environmental changes. Indeed, RNA interference and knock-down studies demonstrated that multiple layers of epigenetic mechanisms are employed in *T. brucei* to regulate genomic functions. Depletion of single epigenetic marks or modifiers (like H3.V, H1, HAT1, HAT2, Sir2rp1) resulted in only partial derepression of gene expression (25,90,111,128,129).

These findings indicate that this early-branching organism relies extensively on a sophisticated epigenetic interface to regulate a genome lacking canonical Pol II transcriptional control elements. In addition, identification of histone epigenetic marks unique to *T. brucei*, like H3 K10 and K11 acetylation in BF cells and H2A C-terminal hyperacetylation, holds tremendous promise for the development of epigenetic therapies to treat the debilitating diseases caused by this parasite.

## DATA AVAILABILITY

Mass spectrometric data generated from this publication have been deposited to the ProteomeXchange Consortium (http://proteomecentral.proteomexchange.org/) via the PRIDE partner repository (130), with the dataset identifier PXD024574, and can be viewed with the open-source Proline Studio spectral viewer available at http://www.profiproteomics.fr/proline/ (131). MNase-ChIP-seq sequence data files analyzed in this study are available from the GEO database under accession number GSE165034.

## ETHICAL CLEARANCE

Use of animals for *in vivo* propagation of BF trypanosomes was approved by the Institutional Ethical Review Committee for the College of Veterinary Medicine, Animal Resources and Biosecurity (SBLS/REC/15/98A; Makerere University, Uganda).

## Supplementary Material

gkac759_Supplemental_Files

## Notes

Present address: Johannes P. Maree, Department of Biology, Villanova University, Villanova, PA 19085, USA.

Present address: Andrey Tvardovskiy, Institute of Functional Epigenetics, Helmholtz Zentrum München, Munich, 85764 Neuherberg, Germany.
