# Supplementary material for: Trypanosoma brucei histones are heavily modified with combinatorial post-translational modifications and mark Pol II transcription start regions with hyperacetylated H2A
Source: Nucleic Acids Res. 2022 Sep 12;50(17):9705–23. doi: 10.1093/nar/gkac759 (PMC9508842; doi:10.1093/nar/gkac759)
Supplement: gkac759_Supplemental_Files [file gkac759_supplemental_files.zip › Supplementary Material.pdf]

SUPPLEMENTARY MATERIAL

Trypanosoma brucei histones are heavily modified with combinatorial post-translational modifications and mark Pol II transcription start regions with hyperacetylated H2A.

Johannes P. Maree[1!], Andrey Tvardovskiy[2#], Tina Ravnsborg[2], Ole N. Jensen[2], Gloria Rudenko[3], Hugh-G Patterton[4*]

[1] Department of Biochemistry, Stellenbosch University, Stellenbosch, 7600, South Africa

[2] Department of Biochemistry and Molecular Biology, VILLUM Center for Bioanalytical Sciences, and Center for Epigenetics, University of Southern Denmark, Odense M, DK-5230 , Denmark

[3] Department of Life Sciences, Imperial College London, London, SW7 2AZ, United Kingdom

[4] Center for Bioinformatics and Computational Biology, Stellenbosch University, Stellenbosch, 7600, South Africa

* To whom correspondence should be addressed. Tel +27 21 8082774; E-mail: hpatterton@sun.ac.za

Current addresses:

[!] Department of Biology, Villanova University, Villanova, PA, 19085, USA

[#] Institute of Functional Epigenetics, Helmholtz Zentrum München, Munich, 85764 Neuherberg, Germany

Keywords:

Trypanosoma brucei, combinatorial histone post-translational modification, transcription, epigenetics, histone hyperacetylation

Supplementary table S1: *T. brucei* histone H2A peptide fragments, alignments, quantitation, MS/MS spectra, and fragmentation tables of newly identified PTMs.

Supplementary table S2: *T. brucei* histone H2A.Z peptide fragments, MS/MS spectra and fragmentation tables of newly identified PTMs.

Supplementary table S3: *T. brucei* histone H2B peptide fragments, alignments, quantitation, MS/MS spectra and fragmentation tables of newly identified PTMs.

Supplementary table S4: *T. brucei* histone H2B.V peptide fragments, MS/MS spectra and fragmentation tables of newly identified PTMs.

Supplementary table S5: *T. brucei* histone H3 peptide fragments, alignments, quantitation, MS/MS spectra and fragmentation tables of newly identified PTMs.

Supplementary table S6: *T. brucei* histone H3.V peptide fragments, MS/MS spectra and fragmentation tables of newly identified PTMs.

Supplementary table S7: *T. brucei* histone H4 peptide fragments, alignments, quantitation, MS/MS spectra and fragmentation tables of newly identified PTMs.

Supplementary table S8: *T. brucei* histone H4.V peptide fragments, MS/MS spectra and fragmentation tables of newly identified PTMs.

Supplementary table S9:

Sheet 1: Data on hyperacetylated H2A antibodies. Details of synthetic peptide sequences used to raise antibodies, indirect ELISAs performed, and antigenic plots.

Sheet 2: ChIP-seq NGS data.

Sheet 3: *T. brucei* PTMs. Summary of histone PTMs described in *T. brucei* in present and past studies.

Sheet 4: Combinatorial PTMs. List of combinatorial PTM patterns identified on *T. brucei* histone isotypes.

Supplementary figures:

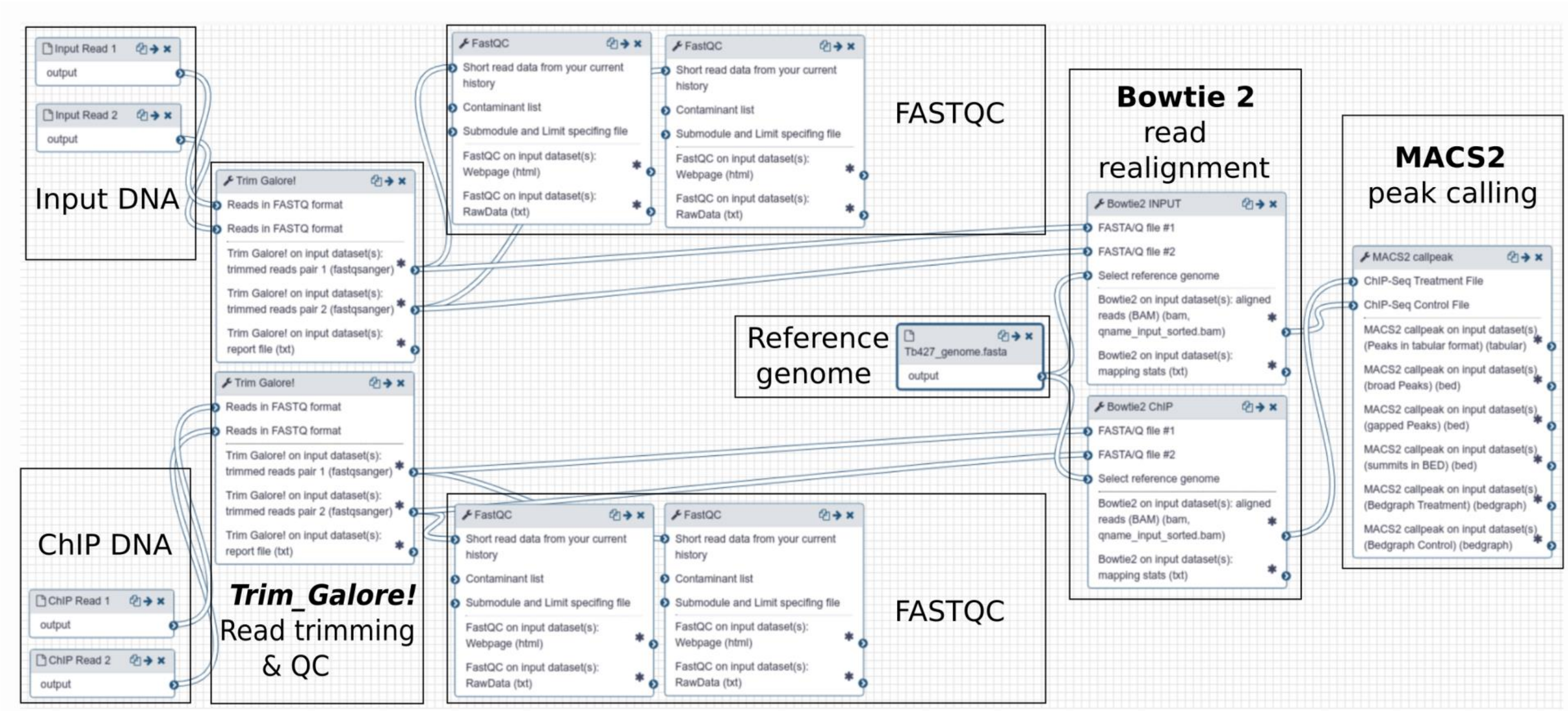

Supplementary figure S1

Custom Galaxy workflow used to process MNase-ChIP-seq data. Input and ChIP reads were trimmed and read quality was confirmed using *Trim_Galore!* and FASTQC. The passed reads were realigned using Bowtie 2, and peaks of enrichment identified using MACS2.

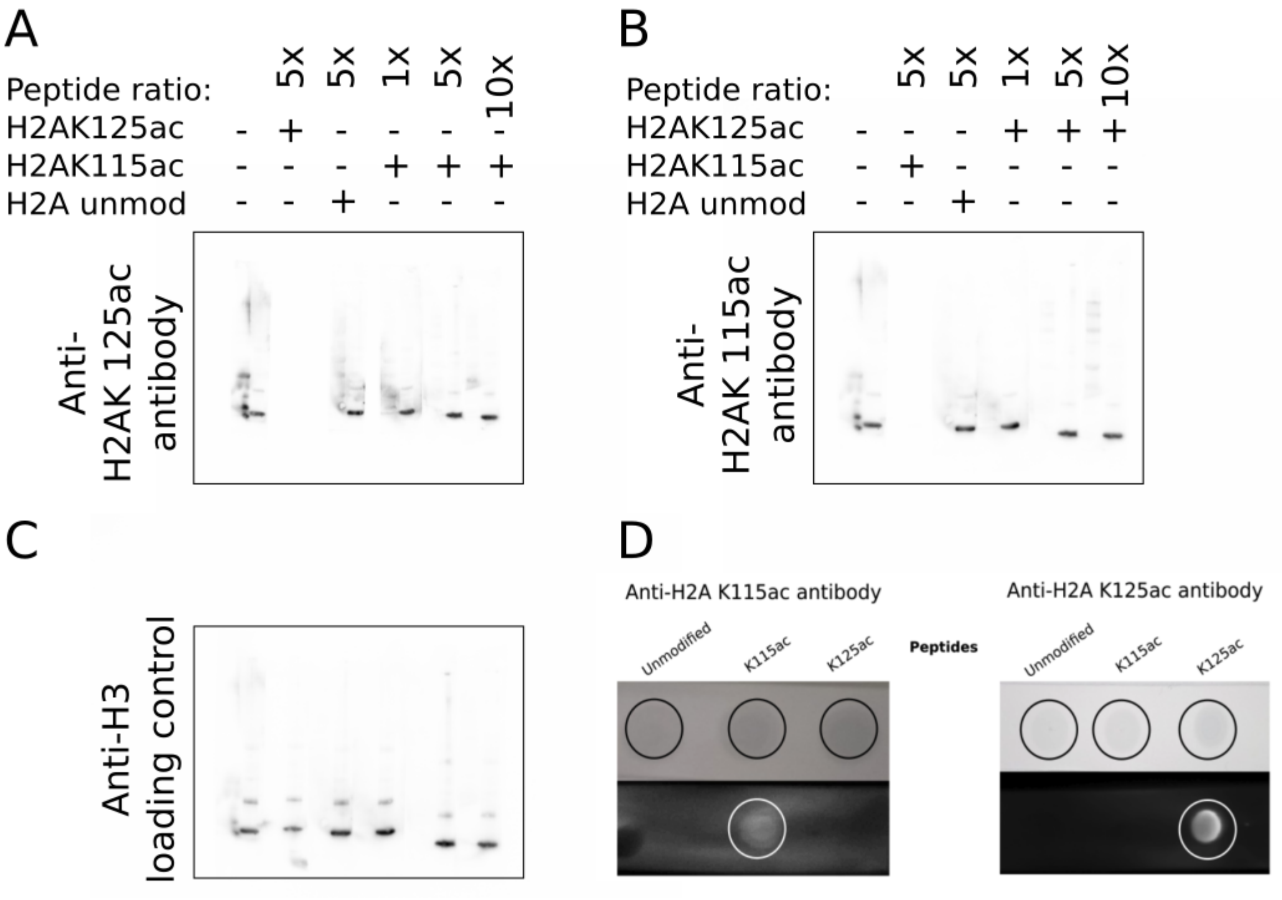

Supplementary figure S2

Validation of antibody specificity. (A) Anti-H2AK125ac antibody and (B) anti-H2AK115ac antibody specificity was evaluated by peptide-based competition assays against unmodified and differentially modified peptides. Text above blots indicate the ratio to which peptides were added to antibodies, with 1x = 1:1 peptide:antibody ratio (2216 molar excess), 5x = 5:1 peptide:antibody ratio (11083 molar excess), and 10x = 10:1 peptide:antibody ratio (22166 molar excess). (C) Anti-histone H3 loading control. (D) Dot-blot against unmodified and differentially modified peptides.

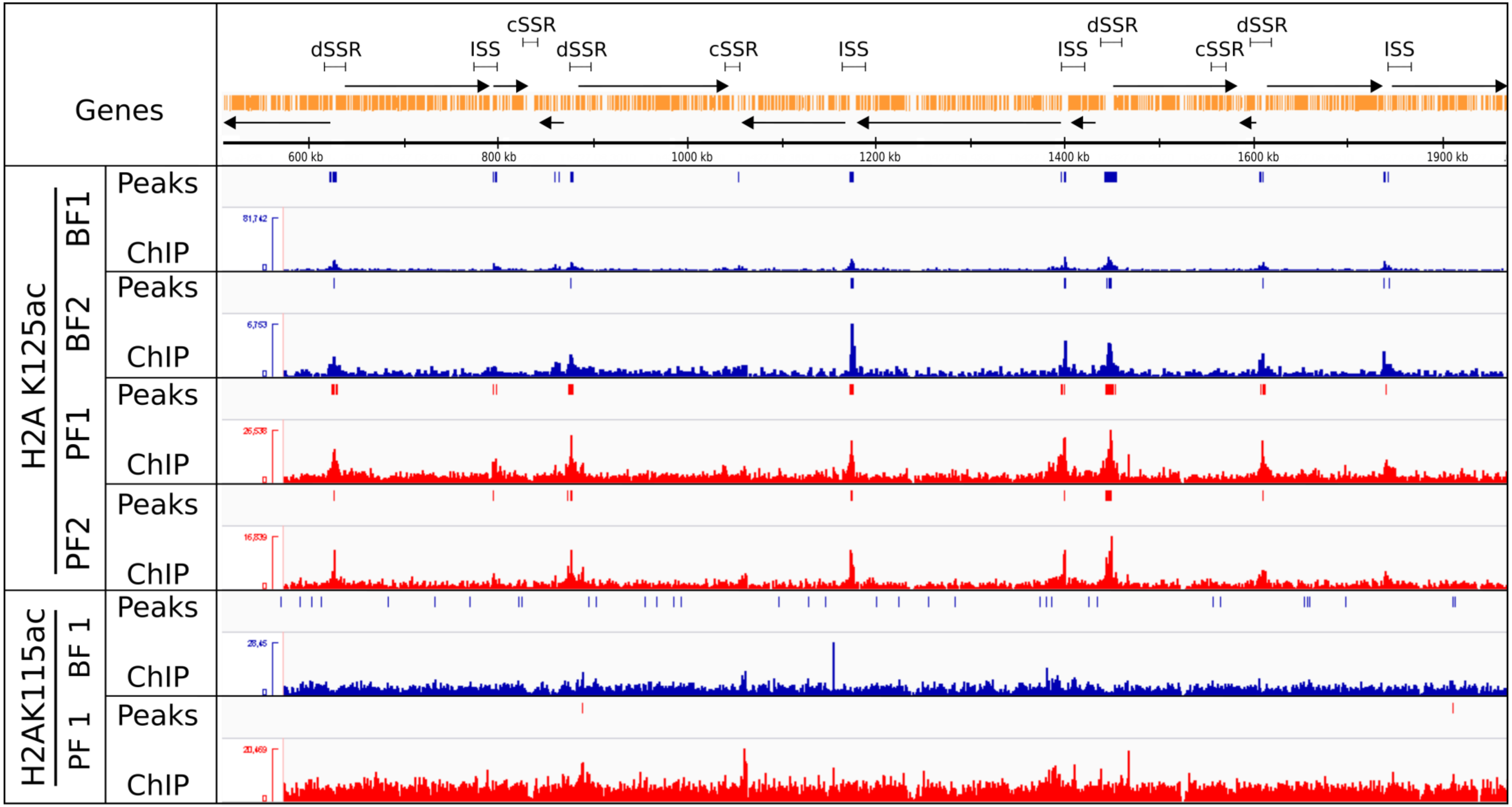

Supplementary figure S3

Distribution of differentially hyperacetylated H2A across different configurations of Pol II transcription boundaries. The genes are shown in the top track in orange, with the direction of Pol II transcription indicated by black arrows. Peaks of enrichment and ChIP profiles are shown for BF (blue) and PF (red) H2AK125ac and H2AK115ac samples on a ~1.35 Mb region on chromosome 8 (523713 - 1866710).

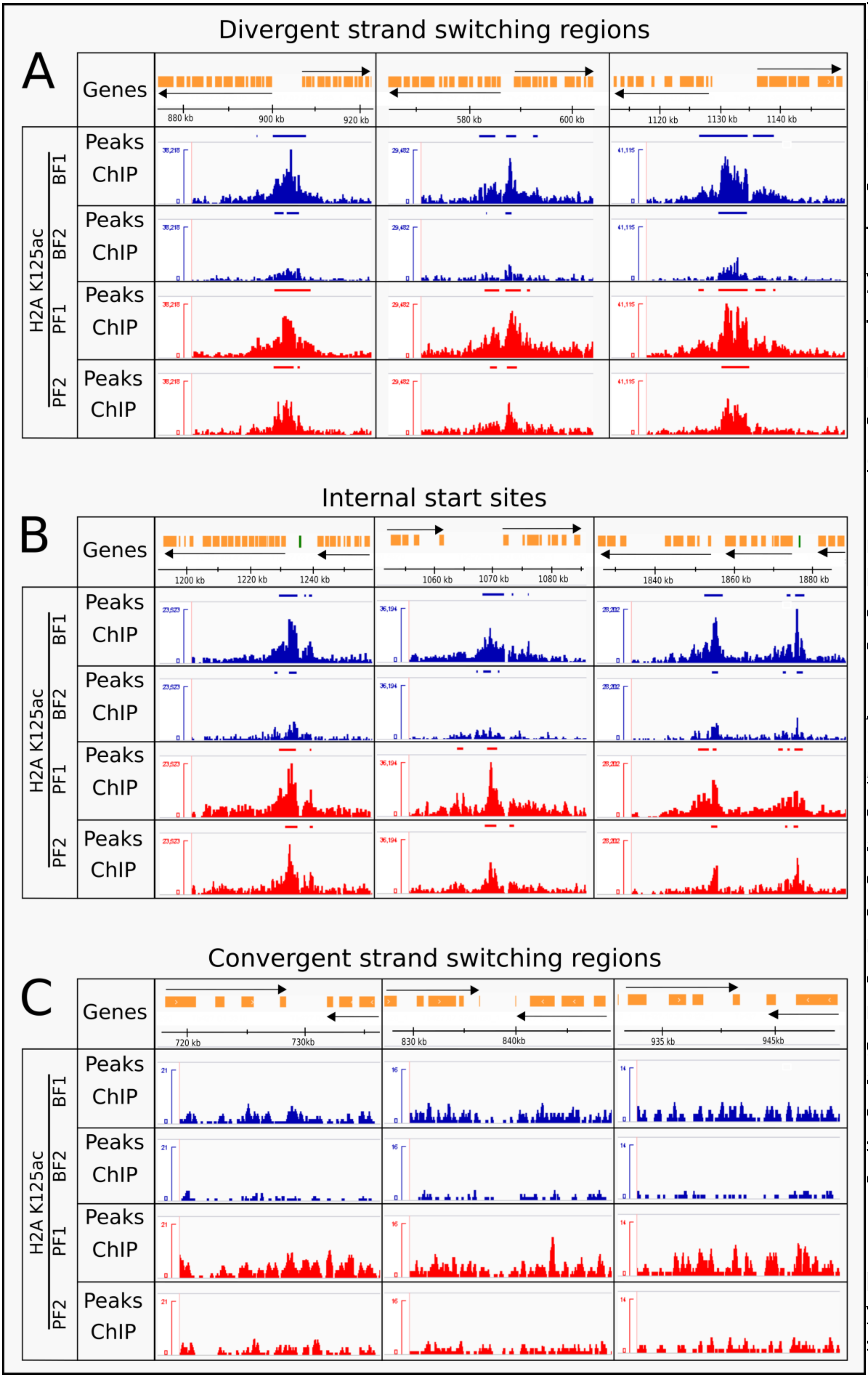

Supplementary figure S4

Enrichment of H2AK125ac at different configurations of Pol II polycistr

onic transcription units. The genes are shown in the top track in orange, with the direction of Pol II transcription indicated by black arrows. Peaks of enrichment and ChIP profiles for BF and PF replicates are indicated in blue and red, respectively. Hyperacetylated histone H2AK125ac at (A) divergent strand switching regions on chromosomes 2 (875630 - 926436), 3 (564441 - 603595), and 6 (664260 - 708851) where Pol II transcription initiates bi-directionally. (B) At internal start regions between two head to tail transcription units on chromosomes 3 (1190501 - 1257322), 9 (1050729 - 1087121), and 11 (1823583 - 1886540) were Pol II transcription terminates and reinitiates. tRNA genes on chromosomes 3 and 11 are indicated with green bars. (C) At convergent strand switching regions on chromosomes 1 (717939 - 735913), 7 (828108 - 849460), and 10 (928753 - 950264) where Pol II transcription terminates.

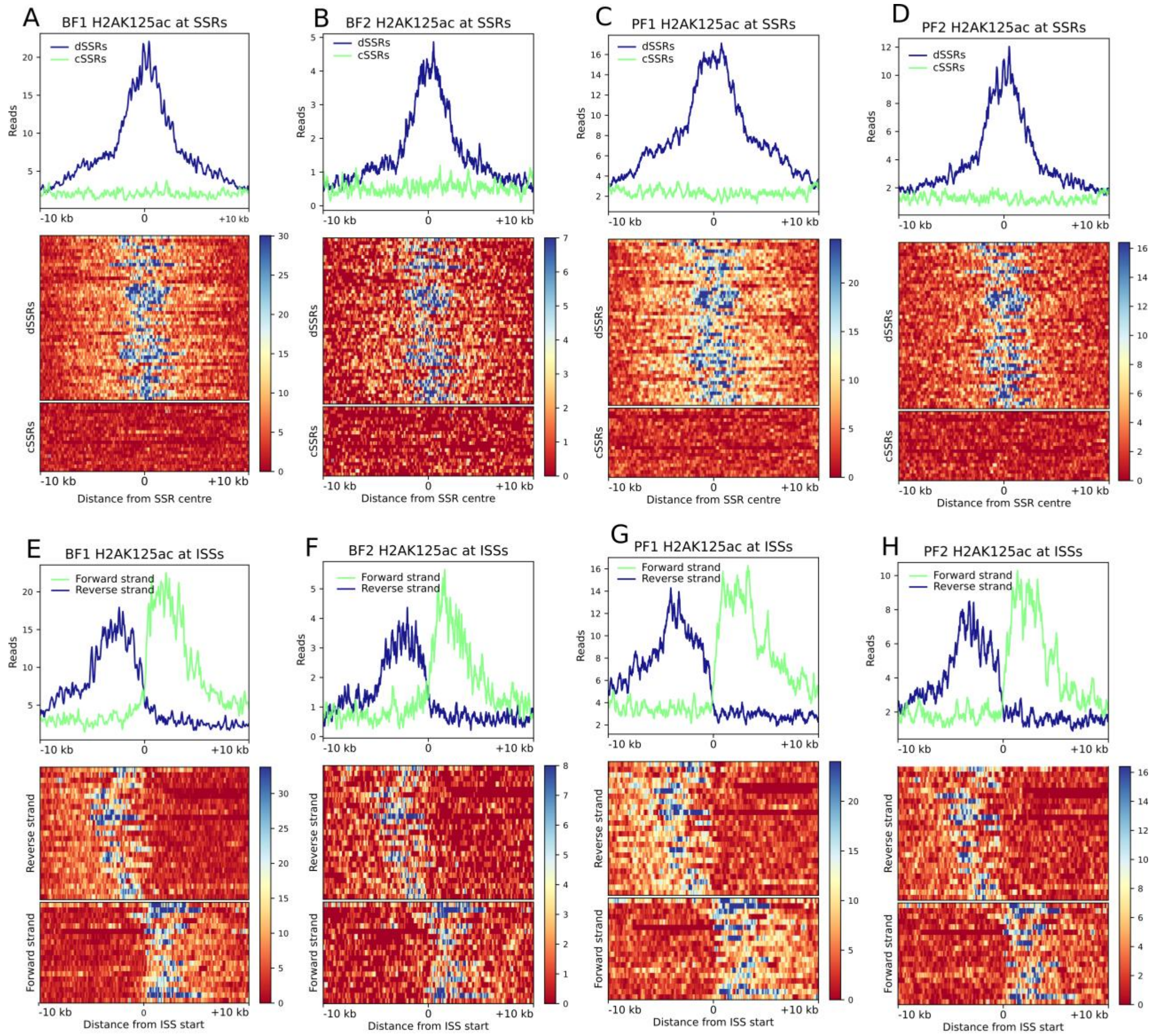

Supplementary figure S5

Heatmaps showing read enrichment profiles around a 20 kb region (10 kb up- and downstream) at different Pol II transcription boundaries. (A) BF1 H2AK125ac at SSRs, (B) BF1 H2AK125ac at ISSs, (C) BF2 H2AK125ac at SSRs, (D) BF2 H2AK125ac at ISSs, (E) PF1 H2AK125ac at SSRs, (F) PF1 H2AK125ac at ISSs, (G) PF2 H2AK125ac at SSRs, (H) PF2 H2AK125ac at ISSs.

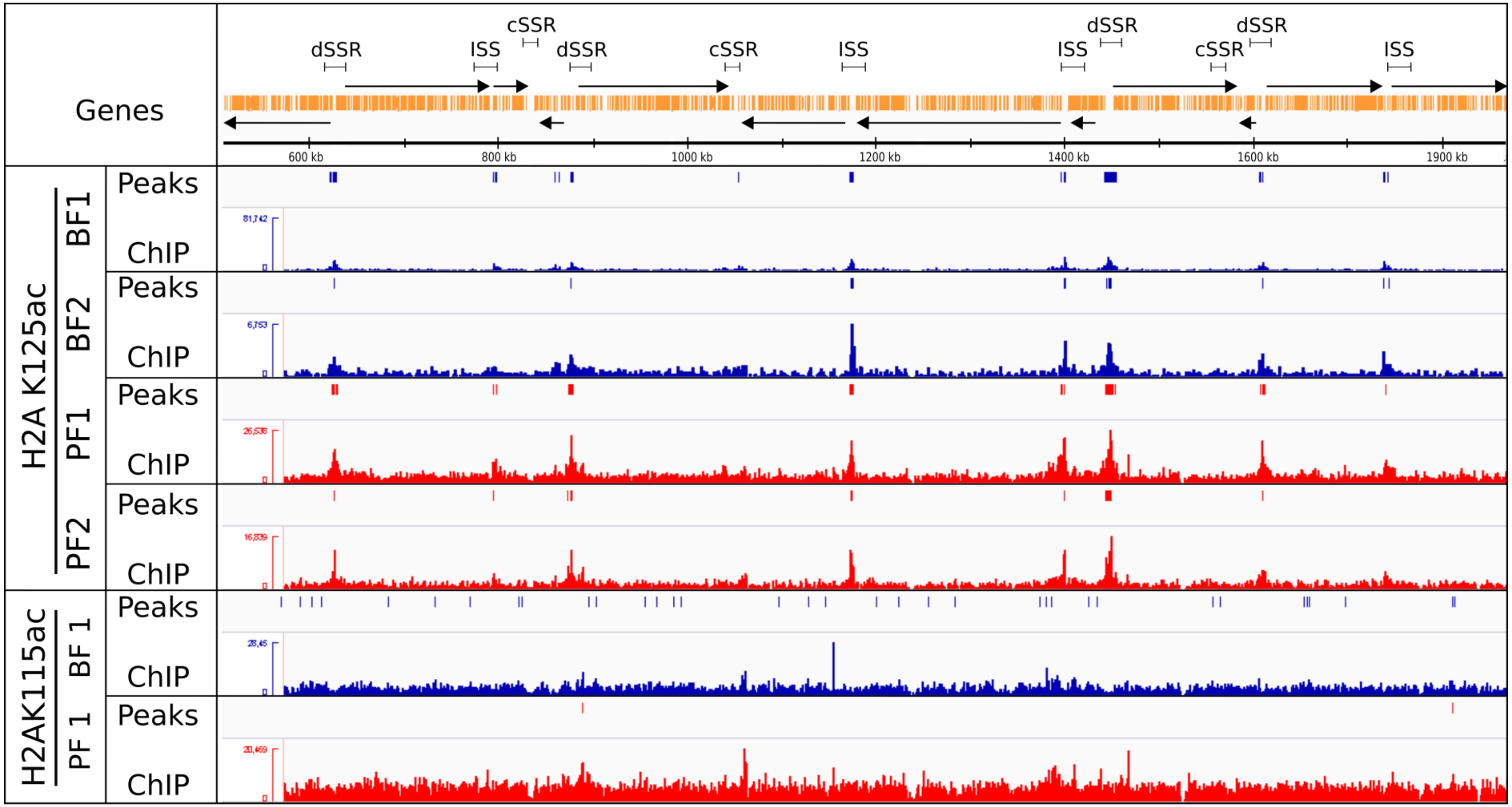

Supplementary figure S6

Enrichment of H2AK125ac-containing nucleosomes on chromosome 11. Regions where Pol II transcription initiates and terminates are indicated above open reading frames, shown in the top track in orange, with the direction of Pol II transcription indicated by black arrows. H2AK125ac and H2AK115ac peaks of enrichment and of ChIP profiles for BF (blue) and PF (red) replicates are shown across a ~800 kb region of chromosome 11 (1617919 - 2422255).

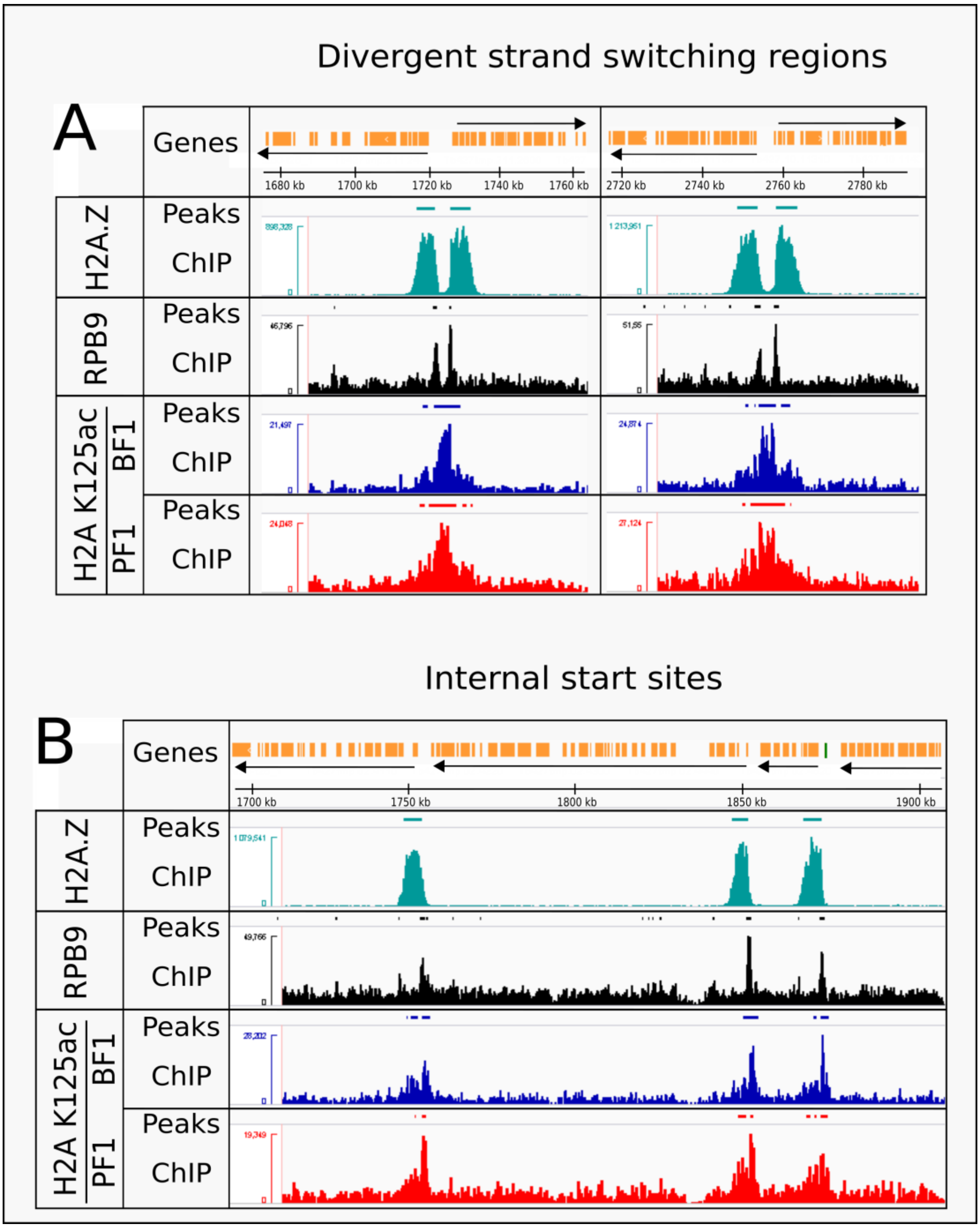

Supplementary figure S7

Distribution of H2A.Z, RPB9, and H2AK125ac at Pol II transcription start sites. H2AK125ac co-localises with H2A.Z and the RPB9 Pol II subunit at Pol II transcription start regions. Open reading frames are shown in orange, with the direction of Pol II transcription indicated by black arrows. Peaks of enrichment and ChIP profiles are shown for H2A.Z (cyan), RPB9 (black), BF H2AK125ac (blue), and PF H2AK125ac (red) at (A) divergent strand switch regions (chromosome 9: 1675592 - 1760797, and chromosome 10: 2717119 - 2787808), and (B) internal start regions between head to tail transcription units (chromosome 11: 1697942 - 1910562) with the presence of a tRNA gene indicated by a green bar.

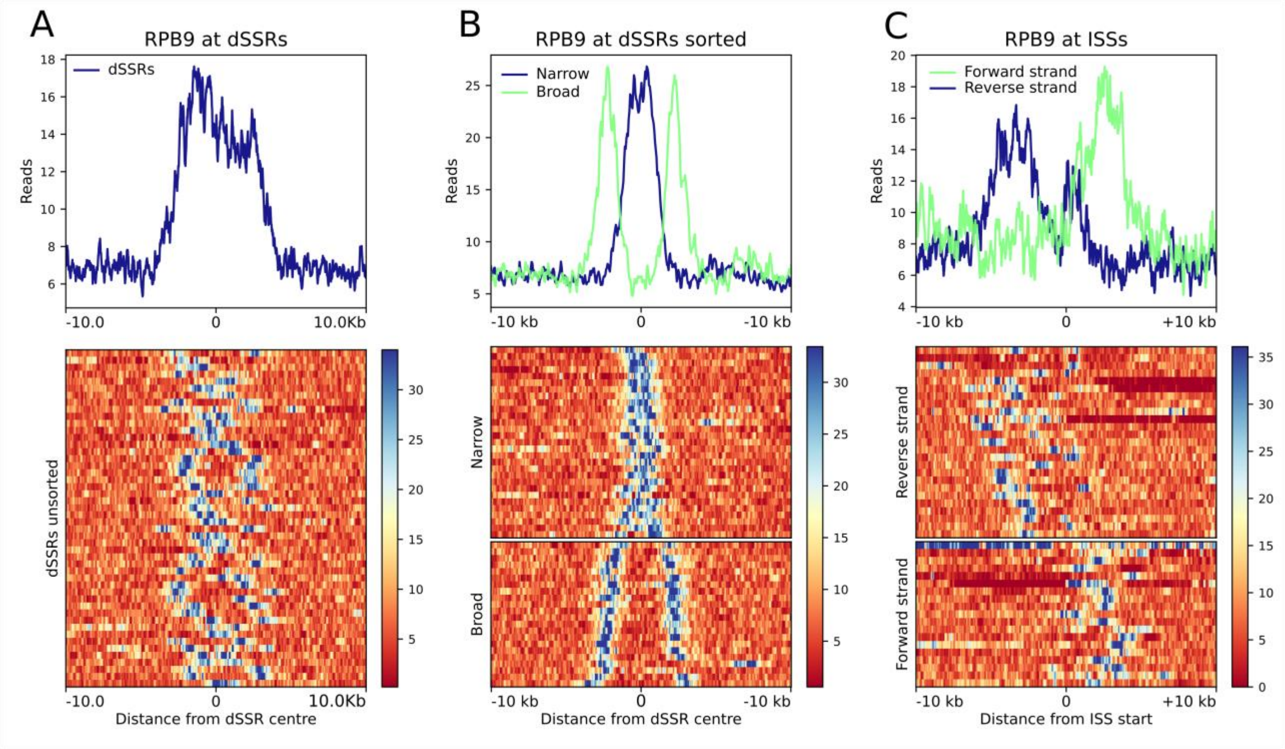

Supplementary figure S8

Heatmaps showing RPB9 read enrichment profiles around a 20 kb region (10 kb up- and downstream) at different Pol II transcription start regions. (A) unsorted RPB9 at dSSRs, (B) sorted RPB9 reads into narrow (≤4000 bp) and broad (>4000 bp) dSSRs, (C) RPB9 at ISSs.

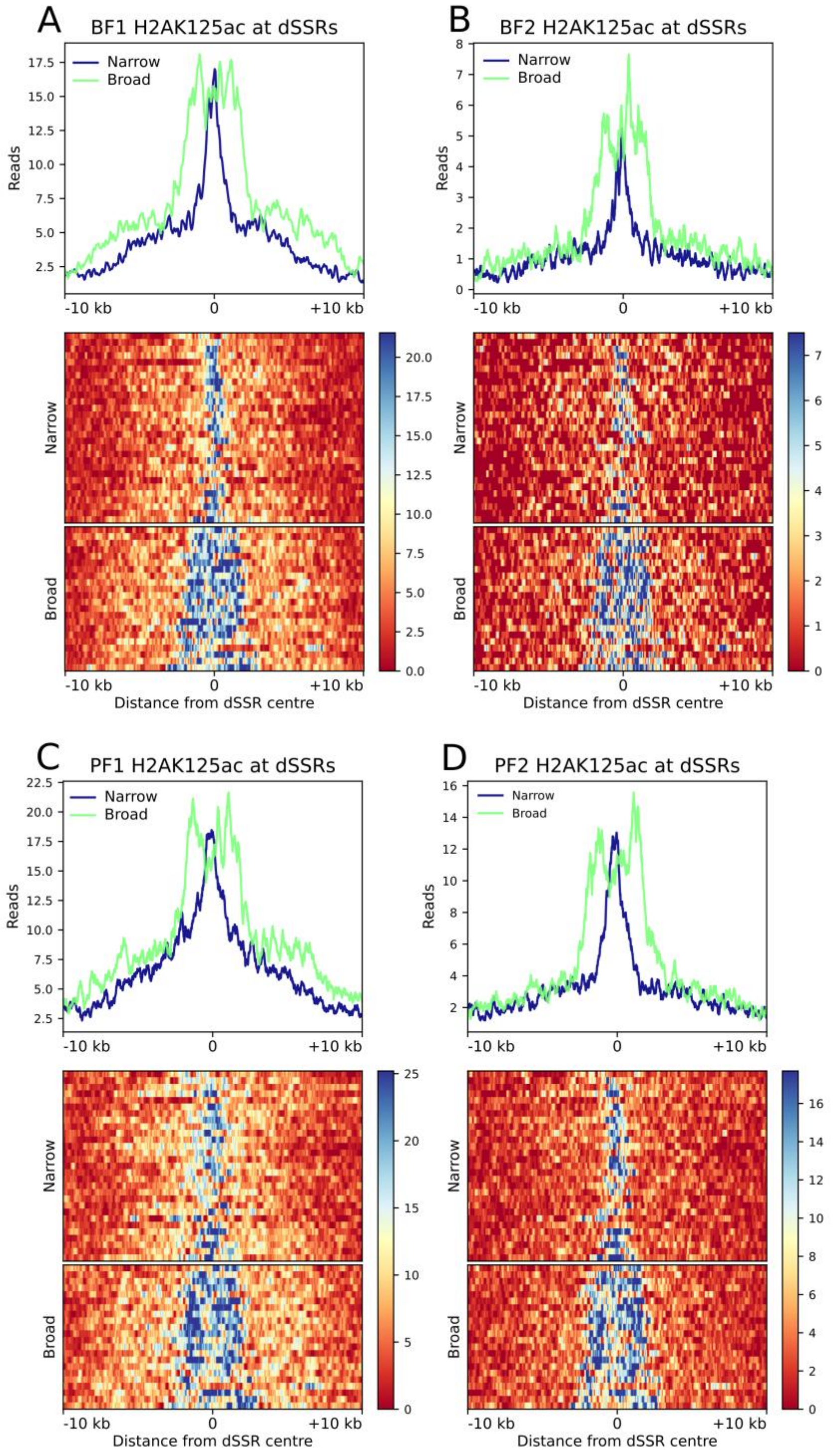

Supplementary figure S9

Heatmaps showing read enrichment profiles around a 20 kb region (10 kb up- and downstream) at narrow (≤4000 bp) and broad (>4000 bp) dSSRs. (A) BF1 H2AK125ac, (B) BF2 H2AK125ac, (C) PF1 H2AK125ac, (D) PF2 H2AK125ac.

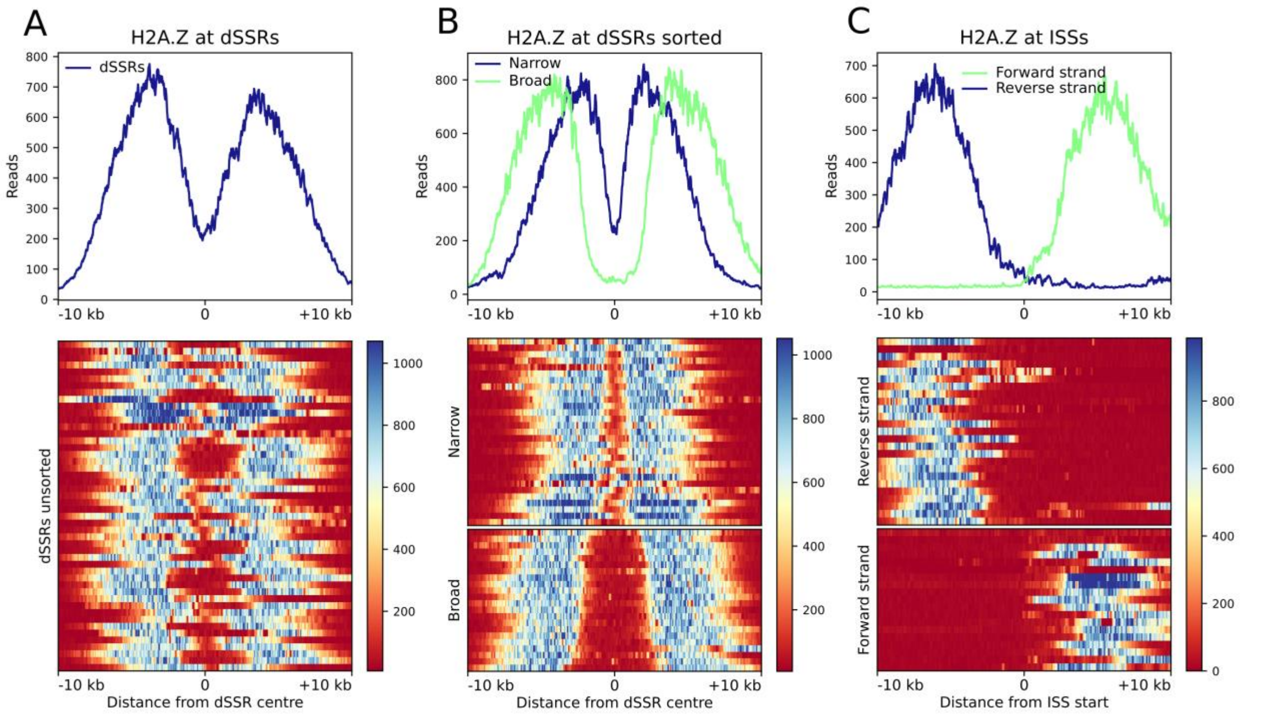

Supplementary figure S10

Heatmaps showing H2A.Z read enrichment profiles around a 20 kb region (10 kb up- and downstream) at different Pol II transcription start regions. (A) unsorted H2A.Z at dSSRs, (B) sorted H2A.Z reads into narrow (≤4000 bp) and broad (>4000 bp) dSSRs, (C) H2A.Z at ISSs.

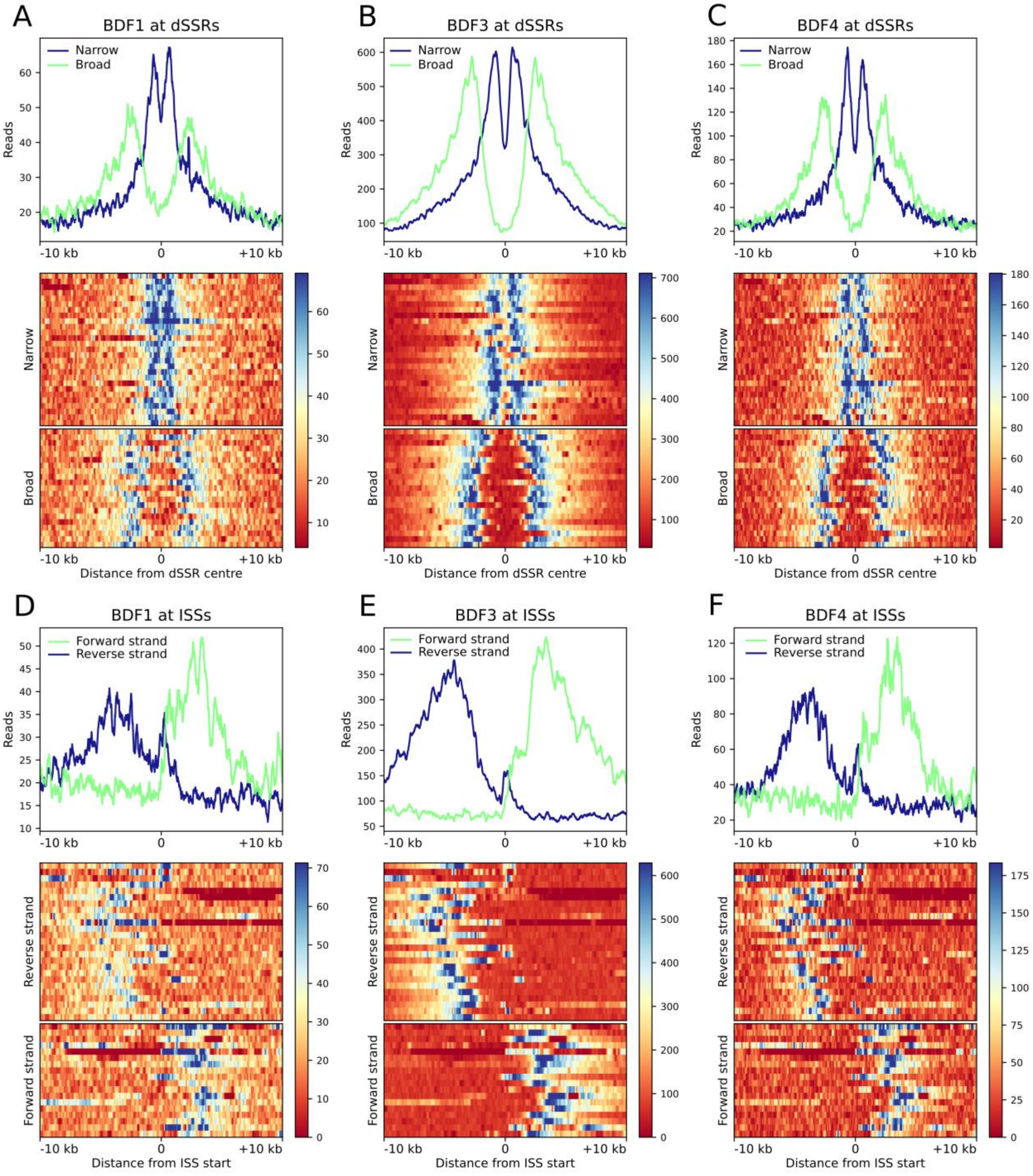

Supplementary figure S11

Heatmaps showing Class I BDFs read enrichment profiles around a 20 kb region (10 kb up- and downstream) at different Pol II transcription start regions. (A) BDF1, (B) BDF3, and (C) BDF4 at narrow and broad dSSRs. (D) BDF1, (E) BDF3, and (F) BDF4 at ISSs.

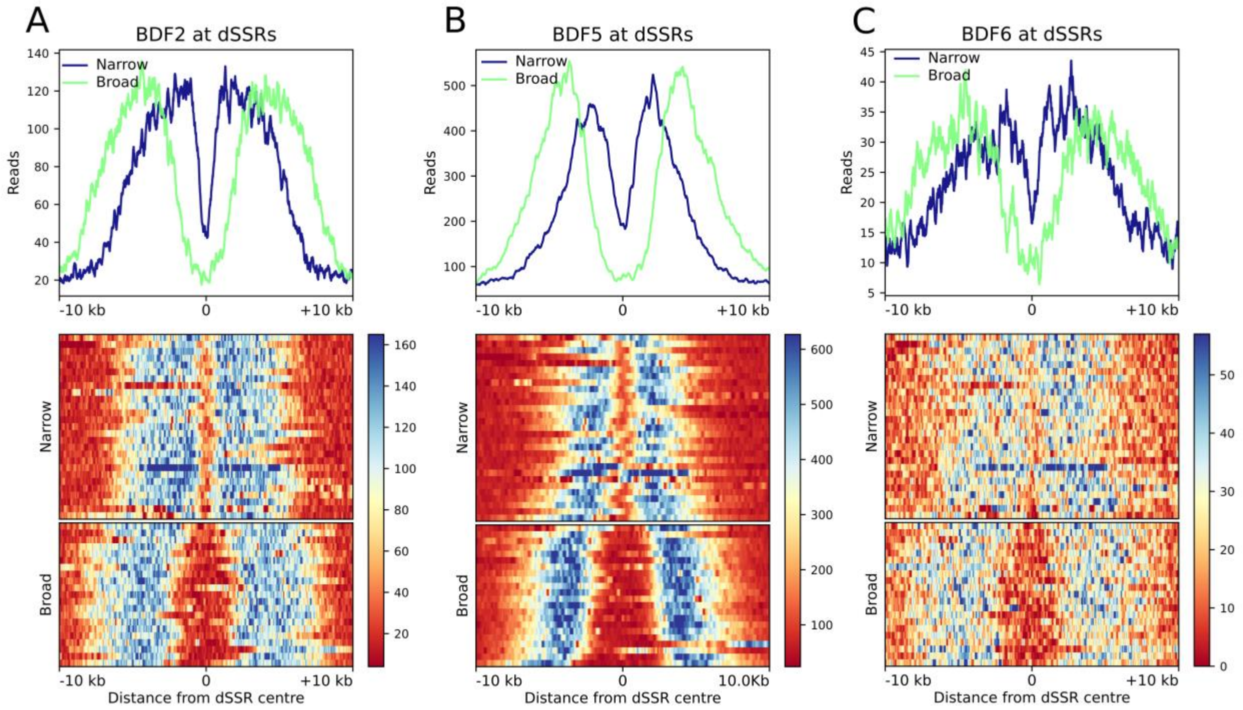

Supplementary figure S12

Heatmaps showing Class II BDFs read enrichment profiles around a 20 kb region (10 kb up- and downstream) at dSSRs. (A) BDF2, (B) BDF5, and (C) BDF6 at narrow and broad dSSRs.

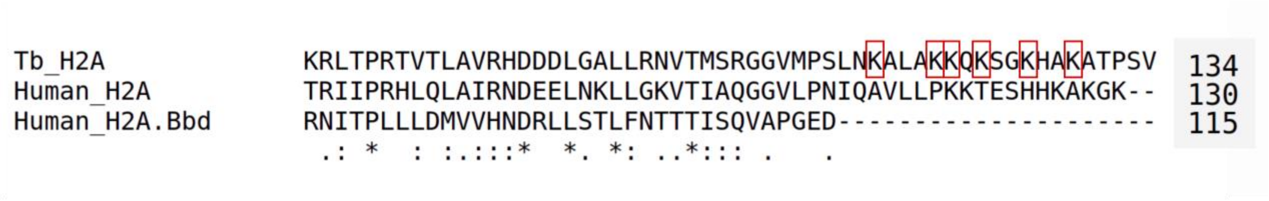

Supplementary figure S13

Multiple sequence alignment of C-terminal tails of *T. brucei* H2A, as well as the *H. sapiens* H2A.1 and H2A.Bbd was performed with Clustal Omega using default parameters (44). Hyperacetylated lysine residues on the *T. brucei* H2A C-terminal tail absent from the truncated human H2A.Bbd tail are indicated by red boxes.
